# Locational distribution of gene functional classes in *Arabidopsis thaliana*

**DOI:** 10.1186/1471-2105-8-112

**Published:** 2007-03-30

**Authors:** Michael C Riley, Amanda Clare, Ross D King

**Affiliations:** 1 Department of Computer Science, University of Wales, Aberystwyth, Penglais, Aberystwyth, Ceredigion, SY23 3DB, Wales, UK

## Abstract

**Background:**

We are interested in understanding the locational distribution of genes and their functions in genomes, as this distribution has both functional and evolutionary significance. Gene locational distribution is known to be affected by various evolutionary processes, with tandem duplication thought to be the main process producing clustering of homologous sequences. Recent research has found clustering of protein structural families in the human genome, even when genes identified as tandem duplicates have been removed from the data. However, this previous research was hindered as they were unable to analyse small sample sizes. This is a challenge for bioinformatics as more specific functional classes have fewer examples and conventional statistical analyses of these small data sets often produces unsatisfactory results.

**Results:**

We have developed a novel bioinformatics method based on Monte Carlo methods and Greenwood's spacing statistic for the computational analysis of the distribution of individual functional classes of genes (from GO). We used this to make the first comprehensive statistical analysis of the relationship between gene functional class and location on a genome. Analysis of the distribution of all genes except tandem duplicates on the five chromosomes of *A. thaliana *reveals that the distribution on chromosomes I, II, IV and V is clustered at *P = *0.001. Many functional classes are clustered, with the degree of clustering within an individual class generally consistent across all five chromosomes. A novel and surprising result was that the locational distribution of some functional classes were significantly more evenly spaced than would be expected by chance.

**Conclusion:**

Analysis of the *A. thaliana *genome reveals evidence of unexplained order in the locational distribution of genes. The same general analysis method can be applied to any genome, and indeed any sequential data involving classes.

## Background

### The locational distribution of genes

It was once thought that the distribution of genes on the chromosomes of eukaryotes was essentially locationally independent, i.e. knowledge of the position of *n *genes on the chromosome does not help you to find the *n *+ 1th gene (just as knowledge of *n *tosses of a fair coin do not help you to predict the *n *+ 1th toss). However, recent studies on the genomes of *Homo sapiens *and *Caenorhabditis elegans *have challenged this view [1-3].

There has been considerable research into the location of genes in prokaryotes since the discovery of the operon in *Escherichia coli *[4]. The genome of *E. coli *has a heterogeneous gene frequency distribution overall [5], but is divided into areas of homogeneous gene frequency [6]. Recent research has found scale invariant correlations [7], convergence of coregulating regions [8], periodicity [9] and strong compositional asymmetries between leading and lagging strands [10]. However, protein synthesis and the structure of the genome in eukaryotes is altogether very different from prokaryotes and consequently the mechanisms affecting gene location in eukaryotes are likely to be very different.

Among the many reasons why genes may not be located independently is the process of genetic mutation by tandem duplication. Tandem duplications (aka tandem repeats) are genetic mutations where a sequence of nucleotides becomes duplicated, with the duplicated sequence lying adjacent to the original sequence. Where tandem duplication extends to duplicating an entire gene, the resulting redundant gene can freely acquire mutations and emerge with a refined or entirely new function [11]. Tandem duplications that include complete genes may produce clusters of identical genes, which become mutated further through subsequent evolution to produce a cluster of similar genes. When considering gene function, it is likely that these genes will belong to the same functional class.

It is still not clear for eukaryotic genomes whether all gene clusters occur simply as a consequence of genetic mutations such as tandem duplication, or whether there is a functional benefit to gene clustering that conveys an evolutionary advantage. We may gain some insight by isolating the known causes of clustering and analysing the gene distributions that remain.

Most research looking into the distribution of genes has focused attention on what are loosely described as clusters [12], and has largely involved analysing histograms of gene loci. In organisms with large genomes, such as *Homo sapiens*, dense clusters of genes are clearly visible in the histograms [13]. However, in organisms with more compact genomes, such as *A. thaliana*, the distribution of genes is more difficult to analyse visually. Therefore a more directly statistical approach is required.

### The *Arabidopsis thaliana *genome

*A. thaliana *is one of the most important model systems for identifying genes and determining their functions and its genome was the first complete genome of a plant to be sequenced. Sequencing of the genome began in 1996 by the Arabidopsis Genome Initiative (AGI)[14] and the results were published by 2000 [15-19]. The length of the genome of *A. thaliana *is now thought to be 157Mbp [20] and there are roughly 25,000 genes encoding proteins with a similar functional diversity to *Drosophila melanogaster*, and *Caenorhabditis elegans*.

Roughly 17% of all genes are arranged in tandem arrays comprising 4140 tandem duplicate genes, most of which are in pairs. Altogether, there are 1528 tandem arrays and the two longest arrays have more than 21 adjacent tandemly repeated genes [14].

Research continues on the genome of *A. thaliana *and of note is a major re-annotation of the entire genome in 2005 [21]. The latest data from many contributors can be found on the TIGR and TAIR websites.

### Methodology overview

In the first part of this study we analyse the locational distribution of all known genes after removing tandem duplicates and genes in the centromeric regions. We use a sliding window analysis where we take the standard deviation of the results as a measure of the degree of clustering and compare with randomly generated sequences of gene locations (see Methods). If tandem duplication and the centromeres are the sole causes of clustering we would expect to obtain locationally independent distributions, which would be statistically related to distributions of genes placed at random on a simulated chromosome. However, the results reveal that, after the removal of the centromeres and tandem repeats, the distribution of all known genes is still locationally dependent.

Further in this study we analyse the locational distribution of genes classified by molecular function. Here we introduce Greenwood's spacing statistic which uses the distances between points or the time between events to give a comparative measure of clustering of those points or events. Low values are indicative of points being evenly spaced apart, whereas high values indicate that points are clustered. Values roughly half way between indicate that the points are distributed at random. We compare the results with those of randomly selected gene locations on the original sequence (see Methods). This gives us a relative measure of how clustered or how evenly spaced the distribution is compared to a locationally independent distribution. We establish the locationally independent distribution using Monte Carlo methods [22] and by using this method we do not need to exclude genes in the centromere, but we do exclude tandem duplicates.

Again, the results reveal that the distribution of molecular functional classes of genes is not locationally independent.

## Results

### Distributions of all genes

The results for the distribution of all genes without tandem duplicates are briefly summarized in table 1, which shows that the genes on all five chromosomes of *A. thaliana *are significantly more clustered than would be expected from a locationally independent distribution.

**Table 1:** Ranking of all genes on each of the chromosomes.

Chr	Rank	Original SD	Mean MC SD	Std Err
1	1000	2.71	2.43	0.054
2	1000	2.67	2.31	0.052
3	957	2.42	2.29	0.051
4	1000	2.74	2.44	0.054
5	1000	2.51	2.19	0.049

Table detailing the ranking, the standard deviation in the distribution of the original genes (Original SD), the mean of 1000 standard deviations from the Monte Carlo simulations (Mean MC SD) and the standard error (Std Err) on all five chromosomes (Chr). The standard deviation gives us a measure of clustering and the difference between Original SD and Mean MC SD divided by Std Err gives us the significance (see main text)

We can use the standard deviation as a measure of clustering, as explained later in the methods section, and we can use the standard error as a measure of the significance of the result. We establish the null hypothesis from the mean standard deviation of 1000 Monte Carlo trials of randomly generated chromosomes. Refering to table 1, we can see that the standard deviation (Original SD) for chromosome I is 2.71 and the mean standard deviation for 1000 Monte Carlo trials of randomly generated chromosomes (Mean MC SD) is 2.43. The standard error for the size of this data set (Std Err) is 0.054. The difference between the standard deviations divided by the standard error is 5.18; i.e. the standard deviation for chromosome I is 5.18 standard errors from the null hypothesis. Any result greater than two standard errors should be considered significant [23] so we can see that this result is very significant.

The standard deviation of the distribution of genes on chromosomes I, II, IV and V ranked 1000 out of 1000 Monte Carlo simulations of a random chromosome. The standard deviations for these chromosomes exceeded 5 standard errors of the mean standard deviation for the Monte Carlo simulations. The standard deviation of chromosome III ranked 957 out of 1000 and had a value of 2.34 standard errors from the mean, which indicates that this result is significant, but there is a slim chance that this distribution could occur by chance.

**Figure 1:**
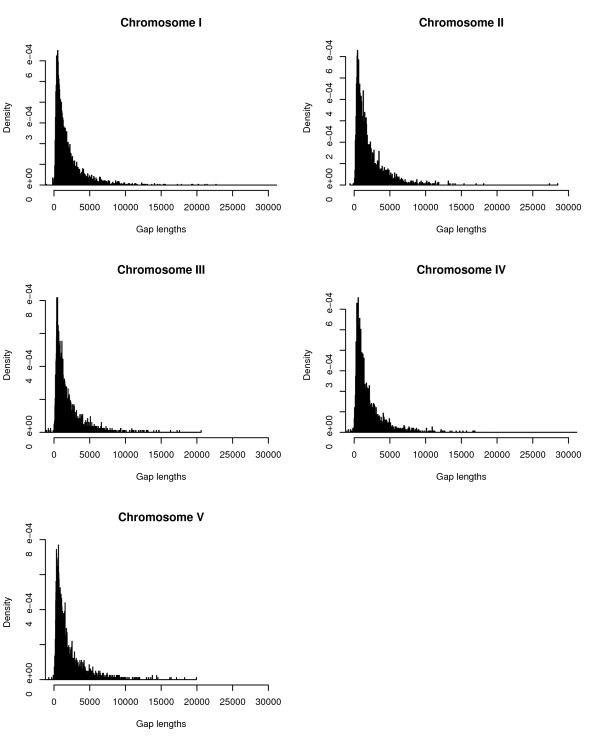
**Probability density plots gap lengths**. Probability density function plots of inter gene gap lengths for all five chromosomes of *A. thaliana*. The curves are not asymptotic to the Y axis; the peak occurs between 300–700 bp.

Further to our analysis of the distribution of the locations of all known genes, the probability density plots of the gap lengths between genes for all five chromosomes are given in figure 1. These plots reveal that the most frequently occurring gap lengths are between 300 and 700 base pairs (bp) on all five chromosomes. Note that the P values given in the abstract are obtained from the ranking thus:-

P=(1000−ranking)+11000     (1)
 MathType@MTEF@5@5@+=feaafiart1ev1aaatCvAUfKttLearuWrP9MDH5MBPbIqV92AaeXatLxBI9gBaebbnrfifHhDYfgasaacH8akY=wiFfYdH8Gipec8Eeeu0xXdbba9frFj0=OqFfea0dXdd9vqai=hGuQ8kuc9pgc9s8qqaq=dirpe0xb9q8qiLsFr0=vr0=vr0dc8meaabaqaciaacaGaaeqabaqabeGadaaakeaacqWGqbaucqGH9aqpdaWcaaqaaiabcIcaOiabigdaXiabicdaWiabicdaWiabicdaWiabgkHiTiabdkhaYjabdggaHjabd6gaUjabdUgaRjabdMgaPjabd6gaUjabdEgaNjabcMcaPiabgUcaRiabigdaXaqaaiabigdaXiabicdaWiabicdaWiabicdaWaaacaWLjaGaaCzcamaabmaabaGaeGymaedacaGLOaGaayzkaaaaaa@4820@

### The locational distribution of functional classes of genes

The full results for the distribution of individual functional classes are listed in tables 1–20 in Additional file 1. The tables are arranged so that each table lists the results for each of the five chromosomes over four levels of the Gene Ontology hierarchy (explained in more detail in the methods section) making 20 tables in total.

The Greenwood statistic of each functional class was compared to 1000 Monte Carlo simulations of a random distribution of the same number of genes as found in each functional class. The average rankings of the Greenwood statistic for all classes in all four levels of the Gene Ontology hierarchy across all five chromosomes are listed in table 2. These show that, in general, the functional classes are more clustered than would be expected from a locationally independent distribution. Furthermore, 12% of functional classes in level 1 were super-clustered having a ranking of 1000 out of 1000.

**Table 2:** Average ranking of each GO level across all five chromosomes.

Level	Ave. ranking (TD removed)	Ave. ranking (all)
1	713	796
2	705	779
3	652	725
4	675	745

Average ranking of all the functional classes analysed with and without tandem duplicates (TD) on all five chromosomes of *A. thaliana *from four levels of the Gene Ontology hierarchy showing that the degree of clustering of the distribution of broadly classified genes is similar to that of the more specific classifications

**Figure 2:**
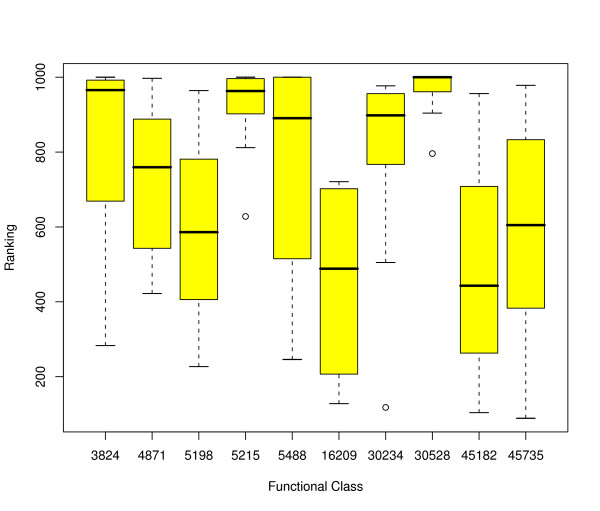
**Distribution of rankings without tandem duplicates**. Distribution of rankings of the functional classes without tandem duplicates at level 1, the ten most general functional classes of the GO hierarchy of both W and C strands across all five chromosomes of *Arabidopsis thaliana*. The labels on the x axis refer to the Gene Ontology classifications described in table 4. The y axis is representative of the relative degree of clustering of genes, where 500 indicates what we would expect if the genes are located at random, above 500 is increasingly clustered and below 500 the genes are increasingly evenly spaced apart. This plot demonstrates that different functional classes have remarkably different degrees of clustering.

For each class there are ten results representing the relative ranking of the Greenwood statistic compared to the null hypothesis, one for each strand on each of the five chromosomes. The individual results can be found in tables 1–5 in the additional file. To better visualize these results for the 10 most populated functional classes at level 1 we used the R statistics software package [24] to create box and whisker plots (aka boxplots) [25] and these are displayed in figure 2. Note that rankings range from 1 to 1000 and a ranking of 500 represent the results we would expect from a locationally independent distribution. Rankings below 500 are increasingly evenly spaced distributions and rankings above 500 are increasingly clustered distributions. The circles represent outliers as interpreted by the default boxplot parameters of the R statistics software.

### Clustered distributions

The functional classifications at level 1 are very broad. It is therefore surprising that there is a marked difference in the degree of clustering among the functional classes. The plots of the genes associated with structural molecule activity (GO:0005198), anti oxidant activity (GO:0016209), translation regulator activity (GO:0045182) and nutrient reservoir classification (GO:0045735) are examples of the distributions that might be expected from these broad classifications, as they show no significant clustering on all five chromosomes for these functional classes. However, most of the functional classes show a high degree of clustering that prevails across all five chromosomes. The plots for genes associated with catalytic activity (GO:0003824), transporter activity (GO:0005215), enzyme regulator activity (GO:0030234), transcription regulator activity (GO:0030528) and binding (GO:0005488) indicate that these functional classes are consistently and very highly clustered throughout the genome.

A number of molecular function subclasses of the five main clustered classes mentioned above are also super-clustered having a ranking of 1000 out of 1000. Referring to the results in the tables in the additional file it can be seen that at level 2 we found five out of ten super-clustered instances of transcription factor activity (GO:0003700), which is a subclass of transcription regulator activity. For the binding class we found 3 out of 10 super-clustered instances of nucleic acid binding (GO:0003676), one of nucleotide binding (GO:0000166), one of protein binding (GO:0005515) and one of lipid binding (GO:0008289) and at level 3 we have one instance of DNA binding (GO:0003677) and one of purine nucleotide binding (GO:0017076). Finally, there are 8 super-clustered subclasses of catalytic activity, which can be found on levels 2, 3 and 4. With catalytic activity class members displaying such a consistency in clustering it was surprising to find that there was one class member at level 4, calcium ion binding (GO:0005509), that had one instance displaying a very evenly spaced distribution with a ranking of 0 out of 1000. Looking at molecular function classes from all levels in the GO hierarchy we found 9 instances of evenly spaced distributions with a ranking of 25 or less out of 1000, which were all members of three of the five main clustered classes, with just two exceptions that belonged to the signal transducer activity class (GO:0004871).

**Figure 3:**
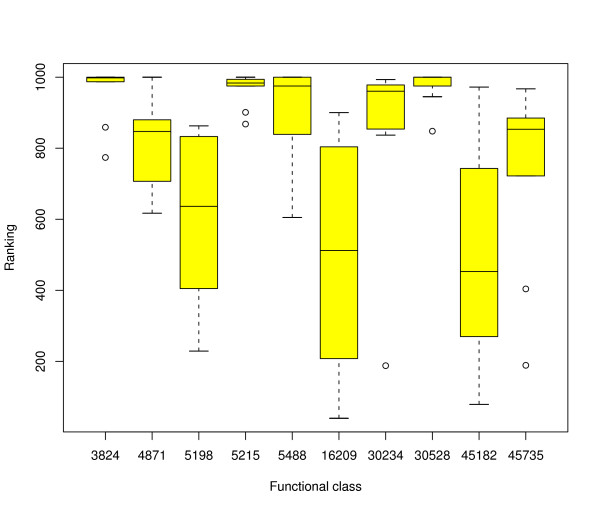
**Distribution of rankings with tandem duplicates**. Distribution of rankings of the functional classes including tandem duplicates at level 1 of the GO hierarchy of both W and C strands across all five chromosomes of *Arabidopsis thaliana*. The labels on the x axis refer to the Gene Ontology classifications. Refer to table 4 for a description of these annotations. This plot is the same as figure 2, but with the tandem duplicates included. This demonstrates that tandem duplicates increase clustering by a small degree in all of the most general functional classes. Note that we found some more specific classes at level 4 that were much less susceptible to tandem duplication (see main text).

We repeated these statistical analyses without removal of tandem duplicates. This resulted in slightly more evidence for clustering but did not affect any major conclusion. The results of this analysis are summarised in figure 3.

### Evenly spaced distributions

We also took a closer look at three specific molecular function classes at level 4 in the GO hierarchy which showed very evenly spaced distributions. These were calcium ion binding activity, G-protein receptor activity and metallopeptidase activity.

Genes associated with calcium ion binding activity (GO:0005509) have a very evenly spaced distribution on the W strand on chromosome IV, having a Greenwood statistic ranking of 0 out of 1000. Closer analysis of these 275 genes shows that 9% of these genes are tandem duplicated compared to the average of 17% for all genes. Using the AGI data for tandem duplicates, 12 tandem arrays were identified, 11 tandem pairs and one tandem triplet. There were no observed tandem duplications on the W strand of chromosome IV.

Genes associated with G-protein coupled receptor activity (GO:0004930) displayed more evenly spaced distributions on both W and C strands on chromosome IV with statistic rankings falling in the lowest 4%. There are 157 genes associated with G-protein receptor activity (GO:0004930) in *A. thaliana*, but only eight tandem duplicates have been identified. Furthermore, there were no tandem duplications on chromosomes II and IV. This class was particularly interesting because we found evenly spaced distributions and no tandem duplications on both strands of chromosome IV. However, there are also no tandem duplications on chromosome II, which has a highly clustered distribution. N.B. the location of G protein coupled receptor activity genes in the human genome are frequently distributed in tandem arrays. Of the 172 genes associated with metallopeptidase activity (GO:0008237) only 10 were tandem duplications with one pair on chromosome I and two pairs and an array of four tandem duplications on chromosome V. This functional class has an average ranking for chromosomes I, II, III and V that is similar to the average ranking for all functional classes, but this class on chromosome IV ranks in the bottom 10% indicating a very evenly spaced distribution. This would indicate that evenly spaced distributions are not necessarily dependent on gene molecular function class.

These three molecular function classes where we have found evenly spaced distributions all have a lower than average frequency of tandem duplications.

## Discussion

We have seen evidence of very high levels of clustering even after the removal of tandem duplicates for half of the number of molecular function classes at level 1. The remaining half showed higher than average levels of clustering compared to the Monte Carlo simulation with just one exception. Throughout the subclass levels 2, 3 and 4 we find both extremes in that there are frequent occurrences of super-clustered distributions and a number of distributions that are more evenly spaced than we would expect. Although it must be considered that the evenly spaced distributions could just possibly have occurred by chance, this seems unlikely and we consider these anomalous distributions to be worthy of more research.

Tandem duplication is thought to be one of the principal mechanisms of gene proliferation and is also thought to be the main cause of clustering. Our results confirm that tandem duplication is a cause of clustering, but is unlikely to be the sole cause. The results of the further analysis of genes associated with G protein coupled receptor activity in *A. thaliana *indicate clearly that tandem duplications are not the only process that generate gene clustering since the distribution of this class on chromosome II is clustered, but contains no tandem duplications.

Another observation regarding tandem duplications is that genes of many individual classes show roughly the same degree of clustering across both strands on all five chromosomes, and this indicates that clustering is in some way dependent on gene molecular function. This may further imply that tandem duplications are gene molecular function dependent.

There are many reasons to expect clustered gene functional distributions as we have already discussed. There is also strong evidence for clustering of structurally related genes in the human genome (using a different statistical approach) [1]. It was therefore surprising to find that some functional classes on some chromosomes were significantly more evenly spaced than would be expected by chance. The evenly spaced distribution of some functional classes would imply something about the nature of genes of that molecular function. We have found that the classes displaying even distributions have fewer than average tandem repeats. It would seem that some gene functional classes do not appear to be so prone to tandem duplication. But since tandem duplication is not the only cause of clustering there is likely to be other factors involved. For example, there maybe an evolutionary advantage in distributing essential genes evenly across the genome.

Other factors affecting the locational distribution of gene functional classes may include the 3 dimensional structure of the chromosome itself. The degree of coiling of the chromatin varies during the life cycle of the cell. When the chromatin is tightly coiled or highly condensed the number of genes physically available for expression is low. More genes are available for expression during the phases required for cell division when the chromatin is decondensed. The chromatin exists in a partially condensed state when a cell has matured. Evidently, in the matured state, less genes are physically available for expression and clearly the genes required for the specific functions of the matured cell must be available. These genes will need to be located in regions of the chromatin that are available for expression and this could lead to both clustering and even spacing. Clustering because essential genes available for expression will occur in the physically accessible areas. Even spacing because the coiling of the chromatin will lead to physically accessible regions having an inherent cyclic nature and essential genes located in these areas will have an evenly spaced distribution on the primary structure of the genome.

## Conclusion

The distribution of all genes and the distribution of individual functional classes of genes in *Arabidopsis thaliana *were found to be more clustered than we would expect from a locationally independent distribution; and although tandem duplications contribute considerably to clustering, they are clearly not the only factor affecting the observed clustered distributions. This result is consistent with the observations of Mayor et al [1] on the distribution of protein structural domains in the human genome. We found three molecular function classes in *A. thaliana *that are significantly more evenly distributed than would be expected from a locationally independent distribution. The mechanism for this evenness is unknown. Both the evidence clustering and the evidence of evenness implies that there are unexplained elements of order in the locational distribution of genes in *A thaliana*.

## Methods

We first analysed the overall gene distribution using a standard statistical technique, then analysed individual functional class distribution using the Greenwood spacing statistic.

### Data

The gene data to be analysed were downloaded from the MIPS website [26] in April 2005. This version of the data was dated 5/5/04. This data was used to extract the base pair (BP) start loci, end loci and BP lengths together with the gene identifiers (IDs). The Gene Ontology molecular function annotations [27] (version 3.230 – 31/3/2005) were downloaded from the TIGR website [28]. From this we extracted lists of gene IDs for each classification [29]. We examined all molecular functional classes that had at least 100 instances across the entire genome with any evidence code. The classes were arranged in levels of increasing specificity. Excluding the obsolete and unknown classes, there are 10 subclasses of the molecular function class. These we have designated as the level 1 classes. The subclasses of these level 1 classes were designated as level 2, and so on for levels 3 and 4. This data was cross referenced with the loci data set to obtain a data set of the loci of each class of genes. This dataset was then used to analyse the distribution of genes on the chromosomes of *A. thaliana*. The molecular functional classes analysed are listed in the additional file together with the results.

### Removal of tandem duplicates

Previous research [1] has demonstrated that tandem duplicates have an impact on the degree of clustering. We were therefore interested in examining how tandem duplicates affect the gene distributions in *A. thaliana*. The AGI have published data on genes thought to be tandem duplicates. They identified these tandem duplicates using BLASTP [30] with a threshold of *E *< 10^-20 ^and one unrelated gene among cluster members was tolerated. By this method they identified 3737 tandem duplicates in 1456 tandem arrays. The latest data on tandem duplicates (release 5.0) was downloaded the TIGR website.

To confirm these results we used BLAST (version 2.2.13) to identify tandem duplicates. We used the same threshold as the AGI of *E *< 10^-20^, but we did not tolerate any unrelated genes within cluster members. Although we identified a similar number of genes to the data downloaded from TIGR, we chose to use the TIGR tandem duplicates data in our further analysis.

All of the genes identified as tandem duplicates were removed from the molecular function class data except for the first gene in each array. A total of 2281 tandem duplicate genes were removed. Clearly, the interval between the remaining gene marking the location of the tandem array and its nearest neighbour is marginally extended, but this has a negligible impact on the results.

### Distribution of all genes

To determine the distribution of all genes on each chromosome of *A. thaliana *we used a sampling window to sum the intergene gap lengths within each of the windows along the entire chromosome minus the centromere (see below). The length of the sampling window was chosen such that the mean for the number of genes in each window is 10. This is a compromise between Poisson asymmetry (see below) from smaller windows and clustering insensitivity from larger windows. Sampling windows were applied sequentially with no overlap. A test example using a 10% overlap gave only a marginal improvement in clustering sensitivity, but at a tenfold cost in processing time.

We used the standard deviation of the results obtained from the above method as a measure of the clustering of the distribution; a high standard deviation would imply a higher degree of clustering. This is because the limiting case would be a constant intergene gap distance (0 standard deviation) which would give an evenly spaced distribution (minimum clustering). To determine how clustered the distributions are, the results are compared to a Monte Carlo simulation [22] of locationally independent events. Each Monte Carlo trial involved creating a 'pseudo-chromosome' by randomly selecting a gene gap length from the original gene data and then randomly selecting a gene length from the original data. Once a gap length or gene length had been selected it was removed from the random selection procedure; such that each datum is selected without replacement. The random selection of gap lengths and gene lengths continues for all the genes in the chromosome being analysed. We are therefore effectively scrambling the locations of the genes. Once the 'pseudo-chromosome' is created, the same statistical analysis is used to obtain the standard deviation of the number of genes in each window. The generation of 'pseudo-chromosomes' in this way is equivalent to a null model that states that all the clustering is due to the known first-order distribution of lengths of genes and gaps between genes. One thousand Monte Carlo trials were taken, producing one thousand values for the standard deviation. The mean value of the standard deviations was recorded and this gives a reliable measure of the clustering of the distribution of genes on a chromosome where the genes are randomly distributed, and so this value can be used for comparison to the original.

As it is well known that genes are depleted within the centromeric regions of eukaryotic chromosomes [31], inclusion of the centromeric region in this analysis would directly indicate clustering. Therefore, since we were more interested in the distribution of genes in the 'main' sequence of the chromosome it was necessary to exclude the data from the centromere of each chromosome. From a gene frequency plot the approximate centre of the centromeres could easily be identified. The centromeric regions were then identified as regions where the average gene frequency for a sampling window of 31,000 bp fell below 6.5 for all contiguous sampling windows about the approximate centre of the centromere. A total of 6200 genes were excluded, which is a fairly large number, but ensures we have excluded all centromeric gene depletion. Details of the beginning and end of each centromere and the genes excluded are shown in table 3.

**Table 3:** Details of genes from the centromeres that were excluded.

Chromosome	Start (Mbp)	End (Mbp)	Genes excluded
1	11.5	18.5	At1g32000 *- *At1g50919
2	0.0	7.2	At2g01050 – At2g16160
3	9.1	17.1	At3g25100 – At3g47090
4	0.0	6.0	At4g00010 – At4g11240
5	5.4	16.9	At5g16500 – At5g42320

Details of the centromeric regions excluded from the analysis showing the start and end locations of the centromeres determined by the method given in the text

### The locational distribution of functional classes of genes

The locational distribution of genes on both W and C strands of each chromosome classified by molecular function was also considered. Mayor et al [1] have previously used a symmetric Poisson distribution to study the related problem of the locational distribution of structural classes of protein in the human genome. This Poisson distribution based approach has the disadvantage that as the expectation or mean decreases the Poisson distribution becomes asymmetric [32]. As some of the classes have less than ten examples on some strands this approach is therefore problematic.

By plotting a series of graphs of the Poisson distribution for a range of expectations from 0 to 10 in increments of 0.5, it can be clearly seen that expectations below 4.5 produce a significantly asymmetric Poisson distribution, resulting in unreliably skewed results. Sampling with an expectation above 4.5 results in there possibly being too few samples for analysis in the smaller data sets such as the molecular function classes at more specific levels in the Gene Ontology hierarchy. The standard error calculated from equation (2) where *n *is the number of samples and *σ *is the standard deviation [33], means that for a set of data of just two or three samples the standard error is thus about 40 – 50%.

seσ=σ2n     (2)
 MathType@MTEF@5@5@+=feaafiart1ev1aaatCvAUfKttLearuWrP9MDH5MBPbIqV92AaeXatLxBI9gBaebbnrfifHhDYfgasaacH8akY=wiFfYdH8Gipec8Eeeu0xXdbba9frFj0=OqFfea0dXdd9vqai=hGuQ8kuc9pgc9s8qqaq=dirpe0xb9q8qiLsFr0=vr0=vr0dc8meaabaqaciaacaGaaeqabaqabeGadaaakeaacqWGZbWCcqWGLbqzdaWgaaWcbaacciGae83Wdmhabeaakiabg2da9maalaaabaGae83WdmhabaWaaOaaaeaacqaIYaGmcqWGUbGBaSqabaaaaOGaaCzcaiaaxMaadaqadaqaaiabikdaYaGaayjkaiaawMcaaaaa@3A7D@

As a general 'rule of thumb' any statistic should only be considered significant if it exceeds two standard errors [23] and consequently, we would be looking for a standard deviation to vary by 80 – 100% to be significant. This is unlikely to be informative and so an alternative approach was considered.

### The Greenwood statistic

The Greenwood statistic is a spacing statistic [34] which has been found to be a good test for the uniformity of a locational distribution, or conversely, how clustered the distribution is. In general, for a given sequence of events in time or space the statistic is given by: -

G(n)=∑i=1n+1Di2     (3)
 MathType@MTEF@5@5@+=feaafiart1ev1aaatCvAUfKttLearuWrP9MDH5MBPbIqV92AaeXatLxBI9gBaebbnrfifHhDYfgasaacH8akY=wiFfYdH8Gipec8Eeeu0xXdbba9frFj0=OqFfea0dXdd9vqai=hGuQ8kuc9pgc9s8qqaq=dirpe0xb9q8qiLsFr0=vr0=vr0dc8meaabaqaciaacaGaaeqabaqabeGadaaakeaacqWGhbWrcqGGOaakcqWGUbGBcqGGPaqkcqGH9aqpdaaeWbqaaiabdseaenaaDaaaleaacqWGPbqAaeaacqaIYaGmaaaabaGaemyAaKMaeyypa0JaeGymaedabaGaemOBa4Maey4kaSIaeGymaedaniabggHiLdGccaWLjaGaaCzcamaabmaabaGaeG4mamdacaGLOaGaayzkaaaaaa@41F5@

where *D*_*i *_represents the interval between events and is a number between 0 and 1 such that the sum of all *D*_*i *_= 1.

Where intervals are given by numbers that do not represent a fraction of the entire sequence, such as the base pair locations of genes, the Greenwood statistic can be modified [35] and is given by

G(n)=∑i=1n+1Xi2Tn2     (4)
 MathType@MTEF@5@5@+=feaafiart1ev1aaatCvAUfKttLearuWrP9MDH5MBPbIqV92AaeXatLxBI9gBaebbnrfifHhDYfgasaacH8akY=wiFfYdH8Gipec8Eeeu0xXdbba9frFj0=OqFfea0dXdd9vqai=hGuQ8kuc9pgc9s8qqaq=dirpe0xb9q8qiLsFr0=vr0=vr0dc8meaabaqaciaacaGaaeqabaqabeGadaaakeaacqWGhbWrcqGGOaakcqWGUbGBcqGGPaqkcqGH9aqpdaWcaaqaamaaqadabaGaemiwaG1aa0baaSqaaiabdMgaPbqaaiabikdaYaaaaeaacqWGPbqAcqGH9aqpcqaIXaqmaeaacqWGUbGBcqGHRaWkcqaIXaqma0GaeyyeIuoaaOqaaiabdsfaunaaDaaaleaacqWGUbGBaeaacqaIYaGmaaaaaOGaaCzcaiaaxMaadaqadaqaaiabisda0aGaayjkaiaawMcaaaaa@45AE@

where

Tn=∑i=1n+1Xi     (5)
 MathType@MTEF@5@5@+=feaafiart1ev1aaatCvAUfKttLearuWrP9MDH5MBPbIqV92AaeXatLxBI9gBaebbnrfifHhDYfgasaacH8akY=wiFfYdH8Gipec8Eeeu0xXdbba9frFj0=OqFfea0dXdd9vqai=hGuQ8kuc9pgc9s8qqaq=dirpe0xb9q8qiLsFr0=vr0=vr0dc8meaabaqaciaacaGaaeqabaqabeGadaaakeaacqWGubavdaWgaaWcbaGaemOBa4gabeaakiabg2da9maaqahabaGaemiwaG1aaSbaaSqaaiabdMgaPbqabaaabaGaemyAaKMaeyypa0JaeGymaedabaGaemOBa4Maey4kaSIaeGymaedaniabggHiLdGccaWLjaGaaCzcamaabmaabaGaeGynaudacaGLOaGaayzkaaaaaa@3FCC@

and *X *represents the base pair length of the interval between start loci of the genes.

The Greenwood statistic is a comparative measure that has a range of values, which is inversely proportional to the number of points being analysed for a sequence of a given length. For example, applying the Greenwood statistic to a sequence of length 55 with eleven evenly spaced points each 5.5 units apart would give a result of 0.1. For a clustered sequence of six points 10 units apart with a cluster of five points 1 unit apart the result is 0.167. The result for a random distribution of 11 points on the sequence will fall somewhere between these values. This can be confirmed empirically.

To determine significance levels for the Greenwood statistic on gene function we used a Monte Carlo approach based on comparing the Greenwood statistic for a particular functional class of genes, with the Greenwood statistic for a thousand simulated chromosomes. These simulated chromosomes are created by randomly selecting the same number of genes as the class under investigation, from any class of genes on the chromosome. In this way we are using the distribution of genes on the existing chromosome as a null model from which we can make a comparison and thereby alleviating the need to exclude genes in the centromeres. By evaluating the Greenwood statistic for one thousand simulated chromosomes we obtained an empirical distribution of the probability of the evenness or clustering of a random distribution. The results of the Greenwood statistic for one thousand simulated chromosomes are arranged by order of value giving us a ranking by which we can compare the Greenwood statistic of the molecular function class under investigation.

To apply the Greenwood statistic accurately to the distances between genes (or intervals on the chromosomes) it is important that the simulated chromosomes generated are exactly the same length as the original chromosome. Also, the interval from the beginning of the chromosome to the start of the first gene and the interval from the end of the last gene to the end of the chromosome must be included in the data. The random selection algorithm utilized Park and Miller's minimal standard congruential multiplicative random number generator [36] ensuring good properties of a random number generator.

## Authors' contributions

MR carried out the computational and statistical studies and drafted the manuscript. AC and RK conceived of the study and helped to draft the manuscript. All authors read and approved the final manuscript.

**Table 4:** Description of GO annotations.

Class No.	Description
GO:0003824	Catalytic activity
GO:0004871	Signal transducer activity
GO:0005198	Structural molecule activity
GO:0005215	Transporter activity
GO:0005488	Binding
GO:0016209	Anti oxidant activity
GO:0030234	Enzyme regulator activity
GO:0030528	Transcription regulator activity
GO:0045182	Translation regulator activity
GO:0045735	Nutrient reservoir

Descriptions of the Gene Ontology annotations used in the boxplots in figure 2 and figure 3

## Supplementary Material

Additional file 1 Locational Distribution of Gene Functional Classes in *Arabidopsis thaliana*. Click here for file
