# Supplementary material for: Locational distribution of gene functional classes in Arabidopsis thaliana
Source: BMC Bioinformatics. 2007 Mar 30;8:112. doi: 10.1186/1471-2105-8-112 (PMC1855069; doi:10.1186/1471-2105-8-112)
Supplement: Additional file 1 — Locational Distribution of Gene Functional Classes in Arabidopsis thaliana. [file 1471-2105-8-112-S1.pdf]

Locational Distribution of Gene Functional Classes in *Arabidopsis thaliana*

Key to tables

The following list is a full description of the column headings in the tables used in this document.

Chr - Chomosome number

Class - A number representing the molecular function classification annotated by Gene Ontology

Std - Strand (Watson/Crick)

Orig. Grnwd - The Greenwood statistic for the distribution of genes on the original chromosomes

MC Grnwd - The mean Greenwood statistic for the distributions of genes from 1000 pseudo-randomly generated chromosomes

SD MC grnwd - The standard deviation for the above statistic

Ranking - The ranking of the original result compared with 1000 simulated results

Examples - The number of examples of molecular function class used

Chr	Class	Std	Orig. Grnwd	MC Grnwd	SD MC grnwd	Ranking	Examples
1	03824	W	0.00461086	0.00345593	0.000321756	1000	872
1	03824	C	0.00428771	0.0037403	0.000265304	940	884
1	04871	W	0.0266987	0.0272012	0.00426267	543	82
1	04871	C	0.0359752	0.0339515	0.00627094	724	67
1	05198	W	0.030008	0.0320846	0.00512607	406	68
1	05198	C	0.034087	0.039007	0.00706869	265	58
1	05215	W	0.013537	0.00962302	0.00102992	997	251
1	05215	C	0.0139946	0.0115202	0.0014391	939	217
1	05488	W	0.00517308	0.00328976	0.00033366	1000	888
1	05488	C	0.00361528	0.00365203	0.000259156	515	916
1	16209	W	0.123493	0.15363	0.0390906	204	12
1	16209	C	0.194071	0.182619	0.0493941	702	10
1	30234	W	0.125975	0.0894086	0.0189708	956	22
1	30234	C	0.102725	0.0681844	0.0140613	977	31
1	30528	W	0.0167562	0.0102226	0.0010794	1000	236
1	30528	C	0.0132615	0.0113704	0.00138724	904	220
1	45182	W	0.0651981	0.0720028	0.0143043	360	28
1	45182	C	0.0775567	0.100828	0.0226898	104	20
1	45735	W	0.282141	0.2374	0.0656962	805	7
1	45735	C	0.109194	0.1462	0.0382068	89	13

Table 1: Results for gene classes taken from level 1 of the Gene Ontology heirarchy on chromosome 1 using TIGR data with the tandem duplications removed

Chr	Class	Std	Orig. Grnwd	MC Grnwd	SD MC grnwd	Ranking	Examples
2	03824	W	0.00493668	0.00518015	0.000450868	283	505
2	03824	C	0.00599414	0.00461907	0.00037109	992	565
2	04871	W	0.0845108	0.0672586	0.0153843	888	32
2	04871	C	0.127841	0.0590474	0.0134551	997	37
2	05198	W	0.0453661	0.045425	0.00901902	587	50
2	05198	C	0.0466727	0.0553937	0.0116024	227	40
2	05215	W	0.0200676	0.0161908	0.00225987	946	147
2	05215	C	0.0302128	0.018107	0.00269904	996	131
2	05488	W	0.00425628	0.00453212	0.000386012	246	608
2	05488	C	0.00436702	0.00445364	0.00034135	449	589
2	16209	W	0.192492	0.197252	0.0474917	522	9
2	16209	C	0.228358	0.212194	0.0536405	703	8
2	30234	W	0.185141	0.123328	0.0302565	956	16
2	30234	C	0.142932	0.0981309	0.0229557	948	21
2	30528	W	0.0393378	0.0172058	0.0023468	1000	137
2	30528	C	0.0558571	0.0189399	0.00290322	1000	125
2	45182	W	0.155136	0.166668	0.0403859	440	11
2	45182	C	0.138527	0.166958	0.0398796	263	11
2	45735	W	0.434175	0.3492	0.101882	846	4
2	45735	C	0.233419	0.192833	0.0493644	833	9

Table 2: Results for gene classes taken from level 1 of the Gene Ontology heirarchy on chromosome 2 using TIGR data with the tandem duplications removed

Chr	Class	Std	Orig. Grnwd	MC Grnwd	SD MC grnwd	Ranking	Examples
3	03824	W	0.00746425	0.00583099	0.000814598	977	643
3	03824	C	0.00672773	0.00540192	0.000715543	954	678
3	04871	W	0.0551221	0.0482816	0.010014	811	49
3	04871	C	0.0960669	0.0520379	0.0117095	996	47
3	05198	W	0.0425475	0.0380615	0.00816041	781	64
3	05198	C	0.055678	0.0371552	0.00804732	964	69
3	05215	W	0.0222488	0.0185479	0.00321253	902	146
3	05215	C	0.0417299	0.0156211	0.00277516	1000	183
3	05488	W	0.00846204	0.00574346	0.000765179	1000	659
3	05488	C	0.00760123	0.00505556	0.000490969	1000	689
3	16209	W	0.126566	0.171025	0.0473813	128	11
3	16209	C	0.149328	0.192084	0.0553073	207	10
3	30234	W	0.149969	0.101144	0.0242922	948	21
3	30234	C	0.106659	0.0906837	0.0234184	812	25
3	30528	W	0.0491048	0.0168493	0.00263517	1000	162
3	30528	C	0.0535272	0.0197254	0.0039783	1000	141
3	45182	W	0.281498	0.225753	0.0659261	849	8
3	45182	C	0.198122	0.129569	0.0353808	956	16
3	45735	W	0.298559	0.277851	0.0772352	689	6
3	45735	C	0.296933	0.16614	0.0488654	978	12

Table 3: Results for gene classes taken from level 1 of the Gene Ontology heirarchy on chromosome 3 using TIGR data with the tandem duplications removed

Chr	Class	Std	Orig. Grnwd	MC Grnwd	SD MC grnwd	Ranking	Examples
4	03824	W	0.0050018	0.00498841	0.000377788	585	500
4	03824	C	0.00538837	0.00519956	0.00043766	669	537
4	04871	W	0.0483991	0.0484439	0.00884644	589	43
4	04871	C	0.0407367	0.0435496	0.00878347	441	51
4	05198	W	0.0552778	0.0554576	0.0102561	585	37
4	05198	C	0.0598803	0.0602493	0.0118551	548	36
4	05215	W	0.0194584	0.0190879	0.00244008	629	117
4	05215	C	0.0258559	0.0175382	0.00277058	980	137
4	05488	W	0.00509508	0.00471058	0.000390767	831	545
4	05488	C	0.00582023	0.00542012	0.000408192	833	514
4	16209	W	0.130003	0.140046	0.0342328	455	13
4	16209	C	0.234765	0.237411	0.0604259	572	7
4	30234	W	0.114209	0.118632	0.0260909	505	16
4	30234	C	0.159594	0.211018	0.0552698	118	8
4	30528	W	0.0216402	0.0173024	0.00228024	961	130
4	30528	C	0.0293432	0.0203354	0.00323235	979	117
4	45182	W	0.149115	0.161546	0.0400584	446	11
4	45182	C	0.148636	0.154753	0.0391065	552	12
4	45735	W	0.234971	0.260521	0.071213	442	6
4	45735	C	0.231433	0.26178	0.0668359	383	6

Table 4: Results for gene classes taken from level 1 of the Gene Ontology heirarchy on chromosome 4 using TIGR data with the tandem duplications removed

Chr	Class	Std	Orig. Grnwd	MC Grnwd	SD MC grnwd	Ranking	Examples
5	03824	W	0.00470506	0.00374654	0.000359107	980	756
5	03824	C	0.00741941	0.00368441	0.000372623	1000	783
5	04871	W	0.0327333	0.0290924	0.00574659	795	81
5	04871	C	0.0362551	0.0398035	0.00895303	422	61
5	05198	W	0.0455819	0.0392262	0.00769359	833	58
5	05198	C	0.0386574	0.0384007	0.00822679	628	63
5	05215	W	0.0144524	0.0130435	0.0022869	812	191
5	05215	C	0.0217139	0.0126649	0.00248345	987	209
5	05488	W	0.00420059	0.00341291	0.000251068	979	862
5	05488	C	0.00400692	0.00338991	0.000343469	948	866
5	16209	W	0.212407	0.244203	0.076583	408	7
5	16209	C	0.122518	0.113782	0.0272106	721	18
5	30234	W	0.140406	0.117355	0.0280249	848	17
5	30234	C	0.10396	0.0925238	0.0203716	767	23
5	30528	W	0.0282562	0.0116901	0.00161739	1000	213
5	30528	C	0.0144269	0.0125376	0.00244378	796	211
5	45182	W	0.111221	0.103659	0.0252331	708	20
5	45182	C	0.0835833	0.0994304	0.0217667	240	21
5	45735	W	0.205942	0.218595	0.0618135	521	8
5	45735	C	0.109241	0.128322	0.033335	320	16

Table 5: Results for gene classes taken from level 1 of the Gene Ontology heirarchy on chromosome 5 using TIGR data with the tandem duplications removed

Chr	Class	Std	Orig. Grnwd	MC Grnwd	SD MC grnwd	Ranking	Examples
1	00166	W	0.0107258	0.00937975	0.00102211	891	258
1	00166	C	0.0129371	0.00863453	0.000971031	1000	301
1	03676	W	0.00883573	0.00583013	0.000557914	1000	441
1	03676	C	0.00627288	0.00602496	0.000603791	719	461
1	03700	W	0.0170799	0.0107792	0.00131015	1000	224
1	03700	C	0.0134604	0.0121163	0.00160345	840	207
1	03735	W	0.0396305	0.0448146	0.00736639	249	48
1	03735	C	0.0382082	0.0501455	0.00941507	46	43
1	03754	W	0.082164	0.07455	0.013918	750	27
1	03754	C	0.0564661	0.0556431	0.0106559	611	39
1	03793	W	0.139116	0.0967526	0.0197183	964	20
1	03793	C	0.100602	0.110653	0.0255398	409	18
1	04386	W	0.154128	0.102456	0.0217551	967	19
1	04386	C	0.0724309	0.0646965	0.0128912	775	33
1	04857	W	0.189537	0.142903	0.0363572	917	13
1	04857	C	0.151728	0.111908	0.0252016	926	18
1	04872	W	0.0542422	0.0447582	0.00787169	901	48
1	04872	C	0.0502293	0.0521483	0.0107102	509	42
1	05386	W	0.0317418	0.0232466	0.00346521	970	98
1	05386	C	0.0274814	0.0261488	0.00443165	710	89
1	05489	W	0.0396485	0.0400886	0.00665184	535	54
1	05489	C	0.0435345	0.0468206	0.00883886	409	47
1	05515	W	0.0237995	0.0216625	0.00311658	798	105
1	05515	C	0.0200567	0.0196699	0.00295315	629	121
1	08135	W	0.0651981	0.0726647	0.0142764	327	28
1	08135	C	0.0775567	0.100526	0.0229796	105	20
1	08289	W	0.0839434	0.117092	0.0263293	29	16
1	08289	C	0.16031	0.145564	0.0377233	736	13
1	08565	W	0.126357	0.0942259	0.0200566	937	21
1	08565	C	0.139498	0.135878	0.0348166	651	14
1	15034	W	0.112825	0.0976714	0.0212671	826	20
1	15034	C	0.0914775	0.111378	0.027302	226	18
1	15075	W	0.0602414	0.0434953	0.00752369	960	49
1	15075	C	0.0680512	0.0493716	0.00901085	959	44
1	15144	W	0.132551	0.125244	0.0285356	688	15
1	15144	C	0.2241	0.0964325	0.0217541	998	21
1	15267	W	0.120784	0.14178	0.0360506	296	13
1	15267	C	0.169791	0.127789	0.0307377	921	15
1	16491	W	0.0150151	0.0148735	0.00193802	622	156
1	16491	C	0.0202212	0.0181181	0.00266202	787	132
1	16740	W	0.0145807	0.00834435	0.000850983	1000	293
1	16740	C	0.0130997	0.00919653	0.00102678	998	279
1	16787	W	0.0119745	0.00811257	0.000836808	1000	303
1	16787	C	0.00959217	0.0081276	0.000885949	938	323
1	16829	W	0.0637395	0.0515665	0.00934176	904	41
1	16829	C	0.056659	0.0441453	0.00802875	923	50
1	16853	W	0.0598458	0.056515	0.010327	708	37
1	16853	C	0.0540784	0.063066	0.0133762	264	34
1	16874	W	0.0488572	0.0579512	0.0107939	169	36
1	16874	C	0.0669417	0.0742597	0.0162767	357	28
1	19825	W	0.097452	0.097467	0.0207345	584	20
1	19825	C	0.0892372	0.109385	0.0248307	200	18

Table 6: Results for gene classes taken from level 2 of the Gene Ontology heirarchy on chromosome 1 using TIGR data with the tandem duplications removed

Chr	Class	Std	Orig. Grnwd	MC Grnwd	SD MC grnwd	Ranking	Examples
2	00166	W	0.0120941	0.0138655	0.00194106	137	174
2	00166	C	0.0145925	0.0138498	0.00194909	719	173
2	03676	W	0.00666209	0.00746938	0.000772488	107	337
2	03676	C	0.00708366	0.00780332	0.000922006	200	326
2	03700	W	0.0394401	0.0181264	0.00274863	999	130
2	03700	C	0.0561825	0.0199386	0.0031745	1000	118
2	03735	W	0.0510885	0.0535256	0.0113382	498	41
2	03735	C	0.0748701	0.0731439	0.0160671	651	29
2	03754	W	0.21383	0.117079	0.0285792	991	17
2	03754	C	0.136785	0.131422	0.0321473	653	15
2	03793	W	0.440455	0.345091	0.0962091	852	4
2	03793	C	0.311615	0.33592	0.0848267	449	4
2	04386	W	0.108975	0.139285	0.0344028	168	14
2	04386	C	0.274744	0.167198	0.0403137	977	11
2	04857	W	0.24701	0.196816	0.0498654	862	9
2	04857	C	0.179946	0.146809	0.0376253	843	13
2	04872	W	0.128379	0.106413	0.0245292	833	19
2	04872	C	0.142208	0.0931953	0.0227478	959	22
2	05386	W	0.0498991	0.0444904	0.00895837	814	50
2	05386	C	0.0595119	0.0399326	0.00800476	975	56
2	05489	W	0.0627677	0.0681163	0.0151927	421	32
2	05489	C	0.0825069	0.0798037	0.0183442	645	27
2	05515	W	0.068128	0.0338404	0.00592966	1000	67
2	05515	C	0.0725999	0.0347042	0.00671814	999	66
2	08135	W	0.155136	0.165098	0.0416062	484	11
2	08135	C	0.138527	0.166002	0.0417856	268	11
2	08289	W	0.192819	0.198526	0.0498995	542	9
2	08289	C	0.311287	0.297822	0.0791541	653	5
2	08565	W	0.242899	0.181196	0.042781	914	10
2	08565	C	0.164953	0.137176	0.0329595	825	14
2	15034	W	0.211733	0.136878	0.0315354	974	14
2	15034	C	0.155726	0.123769	0.0296685	866	16
2	15075	W	0.0647434	0.0740739	0.0162051	320	29
2	15075	C	0.124802	0.0875377	0.0202259	940	24
2	15144	W	0.248186	0.267142	0.0687964	473	6
2	15144	C	0.0932863	0.145517	0.0365054	11	13
2	15267	W	0.121633	0.131592	0.0304631	439	15
2	15267	C	0.273804	0.197277	0.0504965	930	9
2	16491	W	0.0429582	0.0347538	0.00686123	903	66
2	16491	C	0.0394562	0.0321105	0.0059126	902	71
2	16740	W	0.0140019	0.0125648	0.00167913	847	193
2	16740	C	0.0100147	0.0117611	0.00146531	72	207
2	16787	W	0.0109255	0.0126858	0.00169714	88	191
2	16787	C	0.0158142	0.0112878	0.00146346	987	214
2	16829	W	0.0929941	0.0806471	0.018556	801	26
2	16829	C	0.142105	0.0678035	0.014861	997	32
2	16853	W	0.138935	0.139434	0.0340804	597	14
2	16853	C	0.14666	0.101124	0.0243464	940	20
2	16874	W	0.126924	0.0974982	0.0229079	881	21
2	16874	C	0.0955882	0.106161	0.0247493	397	19
2	19825	W	0.211733	0.13867	0.0341782	970	14
2	19825	C	0.133188	0.122226	0.0288378	715	16

Table 7: Results for gene classes taken from level 2 of the Gene Ontology heirarchy on chromosome 2 using TIGR data with the tandem duplications removed

Chr	Class	Std	Orig. Grnwd	MC Grnwd	SD MC grnwd	Ranking	Examples
3	00166	W	0.0150251	0.0128967	0.00184559	892	223
3	00166	C	0.0169452	0.0125978	0.00212673	968	215
3	03676	W	0.0193296	0.00996196	0.00130052	1000	307
3	03676	C	0.0170507	0.00925041	0.00134523	1000	337
3	03700	W	0.0492682	0.0179707	0.00294462	1000	151
3	03700	C	0.0544014	0.0206394	0.00416924	1000	135
3	03735	W	0.0821085	0.0498291	0.0108361	983	47
3	03735	C	0.0614972	0.0492735	0.0109175	876	50
3	03754	W	0.128367	0.0848729	0.0216931	956	26
3	03754	C	0.174207	0.100247	0.0249277	983	22
3	03793	W	0.692063	0.698654	0.1467	520	1
3	03793	C	0.208939	0.255625	0.0750361	320	7
3	04386	W	0.242068	0.120054	0.0304329	995	17
3	04386	C	0.10462	0.125315	0.0337524	299	17
3	04857	W	0.198285	0.162657	0.0453965	829	12
3	04857	C	0.240999	0.154312	0.0420038	956	13
3	04872	W	0.110363	0.0793271	0.0188017	929	28
3	04872	C	0.11981	0.100426	0.0261102	833	22
3	05386	W	0.0666984	0.0535273	0.0124187	880	44
3	05386	C	0.0513102	0.0356247	0.00771152	952	72
3	05489	W	0.0580403	0.0546816	0.0133442	717	43
3	05489	C	0.0924093	0.054163	0.0121947	990	44
3	05515	W	0.0343056	0.0272103	0.00492473	914	93
3	05515	C	0.0306511	0.0271684	0.00599417	795	99
3	08135	W	0.281498	0.222765	0.0624056	855	8
3	08135	C	0.198122	0.12951	0.0329998	963	16
3	08289	W	0.49036	0.362963	0.102025	889	4
3	08289	C	0.165608	0.145065	0.0405642	775	14
3	08565	W	0.193263	0.161309	0.040286	814	12
3	08565	C	0.127085	0.112153	0.0289605	783	19
3	15034	W	0.178703	0.150349	0.0423305	817	13
3	15034	C	0.116053	0.146729	0.0411603	223	14
3	15075	W	0.0983476	0.0977602	0.0252346	604	22
3	15075	C	0.0785358	0.0527191	0.0122656	958	46
3	15144	W	0.27218	0.220952	0.0626298	840	8
3	15144	C	0.23447	0.145341	0.0399757	967	14
3	15267	W	0.113042	0.150383	0.0405381	130	13
3	15267	C	0.248785	0.22389	0.0625603	733	8
3	16491	W	0.0357018	0.0264013	0.00489884	953	96
3	16491	C	0.0299024	0.0299587	0.00636557	597	86
3	16740	W	0.0251587	0.0137798	0.00199968	996	201
3	16740	C	0.0192089	0.0147304	0.00255908	934	195
3	16787	W	0.0136099	0.0118464	0.00167866	878	245
3	16787	C	0.0130666	0.0106046	0.00140157	943	261
3	16829	W	0.105143	0.0592663	0.0139266	990	39
3	16829	C	0.0936301	0.0590276	0.0139352	974	41
3	16853	W	0.153735	0.0841485	0.0200694	992	26
3	16853	C	0.0974768	0.0767759	0.0188336	876	30
3	16874	W	0.159735	0.0964863	0.0236703	979	22
3	16874	C	0.0838723	0.0733686	0.0186595	786	32
3	19825	W	0.113484	0.140931	0.0376668	226	14
3	19825	C	0.120244	0.15608	0.0451583	187	13

Table 8: Results for gene classes taken from level 2 of the Gene Ontology heirarchy on chromosome 3 using TIGR data with the tandem duplications removed

Chr	Class	Std	Orig. Grnwd	MC Grnwd	SD MC grnwd	Ranking	Examples
4	00166	W	0.0162266	0.0141836	0.00153174	891	161
4	00166	C	0.0133603	0.015613	0.00236995	114	156
4	03676	W	0.0100103	0.00865673	0.000769678	946	271
4	03676	C	0.0101189	0.00982037	0.00128182	710	258
4	03700	W	0.0231701	0.0185406	0.00226592	959	120
4	03700	C	0.0312043	0.0216811	0.00358336	974	109
4	03735	W	0.0967996	0.0827177	0.0187241	841	24
4	03735	C	0.114467	0.0891891	0.0198727	899	23
4	03754	W	0.132303	0.113325	0.0270486	816	17
4	03754	C	0.0964538	0.137028	0.0317815	42	14
4	03793	W	0.176233	0.150833	0.0362954	809	12
4	03793	C	0.142791	0.136258	0.0319889	663	14
4	04386	W	0.128754	0.163232	0.0412364	167	11
4	04386	C	0.287412	0.263527	0.0708856	725	6
4	04857	W	0.254984	0.256732	0.0668623	588	6
4	04857	C	0.381647	0.403214	0.107171	506	3
4	04872	W	0.054522	0.0650309	0.0125094	188	31
4	04872	C	0.0553316	0.0608396	0.0122822	385	35
4	05386	W	0.0455547	0.0477932	0.00846805	455	44
4	05386	C	0.0664276	0.0480133	0.00972745	946	46
4	05489	W	0.0509371	0.0535	0.0101575	463	39
4	05489	C	0.0595786	0.0559469	0.0112029	716	39
4	05515	W	0.0498539	0.0395684	0.00643584	937	53
4	05515	C	0.0390918	0.0334943	0.00603047	860	67
4	08135	W	0.149115	0.161563	0.0379221	433	11
4	08135	C	0.148636	0.154628	0.0385351	522	12
4	08289	W	0.152105	0.133703	0.0302301	778	14
4	08289	C	0.227056	0.151784	0.0374692	960	12
4	08565	W	0.16519	0.210706	0.0547764	186	8
4	08565	C	0.14776	0.191958	0.0472044	133	9
4	15034	W	0.199733	0.125047	0.0309225	972	15
4	15034	C	0.226662	0.176564	0.0414653	895	10
4	15075	W	0.0698339	0.0890899	0.0184024	109	22
4	15075	C	0.115726	0.0849851	0.0178984	939	24
4	15144	W	0.183592	0.228376	0.05971	215	7
4	15144	C	0.16285	0.210408	0.0542087	143	8
4	15267	W	0.224898	0.150683	0.0367392	965	12
4	15267	C	0.163498	0.144828	0.0341622	752	13
4	16491	W	0.0225098	0.0257414	0.00372175	156	85
4	16491	C	0.0308966	0.0272853	0.00454996	814	85
4	16740	W	0.0149445	0.0130421	0.00128686	932	173
4	16740	C	0.0166462	0.0157739	0.00238388	730	154
4	16787	W	0.0127713	0.0128543	0.00141491	540	180
4	16787	C	0.0194318	0.0122846	0.00175734	990	202
4	16829	W	0.0712519	0.0895783	0.0188815	135	22
4	16829	C	0.149617	0.0684695	0.0145365	1000	31
4	16853	W	0.173571	0.141627	0.0364694	854	13
4	16853	C	0.0992826	0.0964113	0.0211939	646	21
4	16874	W	0.0814678	0.0932682	0.0201482	294	21
4	16874	C	0.0740432	0.0891636	0.0208811	230	23
4	19825	W	0.200097	0.149703	0.0345069	921	12
4	19825	C	0.227205	0.194447	0.0479438	805	9

Table 9: Results for gene classes taken from level 2 of the Gene Ontology heirarchy on chromosome 4 using TIGR data with the tandem duplications removed

Chr	Class	Std	Orig. Grnwd	MC Grnwd	SD MC grnwd	Ranking	Examples
5	00166	W	0.0171599	0.00963764	0.00178565	987	267
5	00166	C	0.0159983	0.00972401	0.00165804	992	277
5	03676	W	0.00784552	0.006113	0.000996244	933	439
5	03676	C	0.00583458	0.00641422	0.000855619	295	426
5	03700	W	0.0283576	0.0122214	0.00215929	1000	205
5	03700	C	0.0188692	0.0138402	0.00253511	948	191
5	03735	W	0.0748125	0.0550829	0.0118006	926	41
5	03735	C	0.0451762	0.0486394	0.0102796	445	48
5	03754	W	0.076464	0.0608605	0.0129899	883	36
5	03754	C	0.0630745	0.0653014	0.0139664	521	35
5	03793	W	0.104764	0.0720973	0.0164544	958	30
5	03793	C	0.112627	0.0931866	0.0215842	839	23
5	04386	W	0.137231	0.101844	0.0233527	918	20
5	04386	C	0.0921482	0.0935801	0.0217333	538	23
5	04857	W	0.190225	0.19936	0.0555256	540	9
5	04857	C	0.196175	0.200091	0.0538125	553	9
5	04872	W	0.043182	0.041523	0.00849614	661	55
5	04872	C	0.102719	0.0648648	0.0140313	986	35
5	05386	W	0.034037	0.0328198	0.00646322	670	71
5	05386	C	0.0446296	0.0312793	0.00653988	953	79
5	05489	W	0.0853934	0.0452741	0.00922753	998	50
5	05489	C	0.0719073	0.0549533	0.0124121	903	42
5	05515	W	0.0354366	0.019366	0.00366596	996	126
5	05515	C	0.0421771	0.0230558	0.00487118	996	111
5	08135	W	0.111221	0.103082	0.0258864	722	20
5	08135	C	0.0835833	0.102263	0.0263908	246	21
5	08289	W	0.119533	0.102295	0.024839	815	20
5	08289	C	0.314204	0.134596	0.0335815	1000	15
5	08565	W	0.130795	0.117804	0.0282588	751	17
5	08565	C	0.153383	0.160563	0.043315	534	12
5	15034	W	0.13216	0.147834	0.0400977	409	13
5	15034	C	0.113269	0.0893003	0.0210844	883	24
5	15075	W	0.0638186	0.0786285	0.016819	170	27
5	15075	C	0.0835628	0.0510038	0.0114056	979	46
5	15144	W	0.124376	0.120329	0.0310194	660	17
5	15144	C	0.166513	0.186392	0.0522273	426	10
5	15267	W	0.108888	0.148349	0.038589	79	13
5	15267	C	0.0868651	0.0858772	0.0192674	617	25
5	16491	W	0.0260669	0.0209664	0.004027	899	116
5	16491	C	0.0233869	0.020247	0.00396904	830	127
5	16740	W	0.0121352	0.0104459	0.00191743	868	244
5	16740	C	0.0184173	0.0111379	0.00237493	982	241
5	16787	W	0.00999564	0.00903277	0.00114666	815	281
5	16787	C	0.0130848	0.0097178	0.00207321	922	280
5	16829	W	0.0711863	0.0622255	0.0126386	802	35
5	16829	C	0.0479351	0.0577829	0.0121708	211	40
5	16853	W	0.122868	0.0832939	0.0191864	965	25
5	16853	C	0.0695057	0.0861159	0.0201013	187	25
5	16874	W	0.070515	0.0739976	0.0161408	487	29
5	16874	C	0.0662705	0.0739378	0.0158829	371	30
5	19825	W	0.13216	0.146233	0.0362879	407	13
5	19825	C	0.105358	0.0865355	0.0193606	869	25

Table 10: Results for gene classes taken from level 2 of the Gene Ontology heirarchy on chromosome 5 using TIGR data with the tandem duplications removed

Chr	Class	Std	Orig. Grnwd	MC Grnwd	SD MC grnwd	Ranking	Examples
1	03677	W	0.0112648	0.00743769	0.000775259	999	334
1	03677	C	0.00877793	0.00811889	0.00123578	809	335
1	03723	W	0.0432581	0.0414197	0.00742404	691	52
1	03723	C	0.0344318	0.0384559	0.00749782	337	59
1	04888	W	0.061648	0.0592349	0.010758	674	35
1	04888	C	0.0671268	0.0771114	0.0159577	286	27
1	05516	W	0.118875	0.142978	0.0359628	245	13
1	05516	C	0.091567	0.0935347	0.0212433	546	22
1	08026	W	0.191664	0.132707	0.0312037	937	14
1	08026	C	0.0921932	0.100466	0.0222138	409	20
1	08135	W	0.0651981	0.0733003	0.0151423	320	28
1	08135	C	0.0775567	0.10067	0.0224354	92	20
1	08233	W	0.0233759	0.0267963	0.00391656	178	83
1	08233	C	0.0206304	0.0253335	0.00434446	70	93
1	08324	W	0.0892873	0.0643345	0.0122961	957	32
1	08324	C	0.0985978	0.0693797	0.0156099	955	31
1	08509	W	0.122651	0.118825	0.0287947	649	16
1	08509	C	0.251104	0.155078	0.0392027	974	12
1	15036	W	0.0889079	0.0901678	0.0196119	564	22
1	15036	C	0.109883	0.110495	0.0246957	575	18
1	15268	W	0.160893	0.165289	0.0441906	555	11
1	15268	C	0.169791	0.13006	0.0337903	888	15
1	15290	W	0.0543088	0.0471399	0.00858893	863	45
1	15290	C	0.0558487	0.0449885	0.00837621	913	49
1	15399	W	0.071959	0.0500326	0.00921786	975	42
1	15399	C	0.0715982	0.0730087	0.0156018	552	29
1	16614	W	0.109443	0.0727909	0.0138139	981	28
1	16614	C	0.0704166	0.0725814	0.0154577	525	29
1	16684	W	0.101995	0.1429	0.0357926	46	13
1	16684	C	0.239059	0.24122	0.0747899	618	7
1	16705	W	0.084949	0.0972554	0.0203405	299	20
1	16705	C	0.190129	0.145093	0.0353034	905	13
1	16741	W	0.0598148	0.0900472	0.0191944	3	22
1	16741	C	0.0751853	0.0883928	0.0193237	240	23
1	16746	W	0.136794	0.107688	0.0236941	892	18
1	16746	C	0.091873	0.0729661	0.0151366	901	29
1	16757	W	0.0667384	0.0439505	0.00771198	981	49
1	16757	C	0.0464788	0.0409976	0.00761115	811	54
1	16765	W	0.107635	0.0975849	0.0212669	767	20
1	16765	C	0.109202	0.129399	0.0330854	284	15
1	16772	W	0.0199833	0.0143497	0.0020718	965	164
1	16772	C	0.0222955	0.0169544	0.0026394	960	143
1	16788	W	0.0373592	0.0252128	0.00383814	989	89
1	16788	C	0.0220473	0.024448	0.00390301	272	96
1	16798	W	0.0545562	0.051458	0.00898214	705	41
1	16798	C	0.0345892	0.0385588	0.00729644	332	58
1	16817	W	0.0340734	0.0316031	0.00527004	730	70
1	16817	C	0.0434497	0.0301946	0.00521785	976	76
1	16830	W	0.288981	0.2113	0.0572581	907	8
1	16830	C	0.114107	0.121327	0.0291212	481	16
1	16835	W	0.114373	0.119055	0.0280642	530	16
1	16835	C	0.11854	0.106071	0.0268915	780	19
1	16879	W	0.0965149	0.0946107	0.0201971	620	21
1	16879	C	0.0932646	0.145352	0.037394	6	13
1	17076	W	0.0114333	0.0102652	0.00112816	868	234
1	17076	C	0.0131537	0.00902253	0.00102379	996	285
1	46872	W	0.024028	0.0180166	0.00237599	978	127
1	46872	C	0.0230331	0.0180724	0.00275136	952	133
1	46873	W	0.182189	0.153105	0.0401475	829	12
1	46873	C	0.113717	0.110653	0.0287141	638	18

Table 11: Results for gene classes taken from level 3 of the Gene Ontology heirarchy on chromosome 1 using TIGR data with the tandem duplications removed

Chr	Class	Std	Orig. Grnwd	MC Grnwd	SD MC grnwd	Ranking	Examples
2	03677	W	0.0104227	0.0103567	0.00121916	617	236
2	03677	C	0.0121405	0.0111161	0.0014223	815	218
2	03723	W	0.0346826	0.0377351	0.00711278	388	60
2	03723	C	0.0314191	0.0356429	0.00747756	298	64
2	04888	W	0.186446	0.136962	0.0323555	923	14
2	04888	C	0.189256	0.129371	0.0332084	949	15
2	05516	W	0.174792	0.156617	0.0385985	738	12
2	05516	C	0.173693	0.146776	0.0366692	800	13
2	08026	W	0.13553	0.157857	0.0403405	327	12
2	08026	C	0.313679	0.233308	0.0580832	907	7
2	08135	W	0.155136	0.167164	0.0429292	452	11
2	08135	C	0.138527	0.165157	0.0399058	286	11
2	08233	W	0.0456441	0.0501534	0.0101662	387	44
2	08233	C	0.0329689	0.0303633	0.00575822	761	77
2	08324	W	0.0783946	0.0940081	0.0219553	240	22
2	08324	C	0.142701	0.13091	0.0331694	728	15
2	08509	W	0.222295	0.215237	0.0573555	652	8
2	08509	C	0.258681	0.211388	0.0567755	842	8
2	15036	W	0.538734	0.265629	0.0691196	995	6
2	15036	C	0.182638	0.211328	0.0530256	336	8
2	15268	W	0.121633	0.12867	0.0291115	478	15
2	15268	C	0.274044	0.211519	0.054928	890	8
2	15290	W	0.105731	0.0943084	0.0227199	758	22
2	15290	C	0.0749605	0.0676848	0.0152143	752	32
2	15399	W	0.110104	0.0870072	0.0192825	894	24
2	15399	C	0.10166	0.0968783	0.0227527	672	21
2	16614	W	0.145478	0.135955	0.031394	690	14
2	16614	C	0.167532	0.145675	0.0346953	777	13
2	16684	W	0.232707	0.21529	0.0546774	708	8
2	16684	C	0.223316	0.196067	0.0473663	780	9
2	16705	W	0.240199	0.237971	0.0627918	608	7
2	16705	C	0.195492	0.166382	0.0439648	806	11
2	16741	W	0.20979	0.196969	0.049474	685	9
2	16741	C	0.160775	0.168885	0.043349	494	11
2	16746	W	0.104523	0.130026	0.0325353	196	15
2	16746	C	0.0890638	0.101596	0.0247075	363	20
2	16757	W	0.0688639	0.0692211	0.0153597	598	31
2	16757	C	0.114446	0.0736175	0.0176818	973	29
2	16765	W	0.194296	0.156408	0.0404844	856	12
2	16765	C	0.287522	0.233806	0.0585521	856	7
2	16772	W	0.0176957	0.0202128	0.0032316	196	117
2	16772	C	0.0166322	0.0190175	0.00289368	190	124
2	16788	W	0.0323267	0.0327083	0.00606734	576	70
2	16788	C	0.0282482	0.0312208	0.00577268	347	75
2	16798	W	0.0909692	0.089414	0.0202567	627	23
2	16798	C	0.136232	0.0777297	0.0177416	986	27
2	16817	W	0.0403907	0.0417495	0.00835943	534	54
2	16817	C	0.0561158	0.0573333	0.0135947	570	39
2	16830	W	0.142254	0.195474	0.0489293	102	9
2	16830	C	0.193914	0.180522	0.0444134	706	10
2	16835	W	0.198481	0.145977	0.0347089	927	13
2	16835	C	0.204152	0.147749	0.038601	923	13
2	16879	W	0.203086	0.138168	0.0323754	958	14
2	16879	C	0.104247	0.116908	0.0281578	390	17
2	17076	W	0.0124449	0.0150802	0.00213807	42	159
2	17076	C	0.0149238	0.0148545	0.00211246	597	160
2	46872	W	0.0562068	0.0275346	0.00497476	999	84
2	46872	C	0.0503996	0.031712	0.006277	982	73
2	46873	W	0.20553	0.180018	0.0451437	768	10
2	46873	C	0.239438	0.212352	0.0505511	749	8

Table 12: Results for gene classes taken from level 3 of the Gene Ontology heirarchy on chromosome 2 using TIGR data with the tandem duplications removed

Chr	Class	Std	Orig. Grnwd	MC Grnwd	SD MC grnwd	Ranking	Examples
3	03677	W	0.0263911	0.012224	0.00165541	1000	237
3	03677	C	0.0196053	0.0123025	0.00198502	996	241
3	03723	W	0.0741158	0.0598415	0.0138504	862	38
3	03723	C	0.101448	0.049219	0.011411	996	49
3	04888	W	0.184884	0.142813	0.038696	874	14
3	04888	C	0.1849	0.137107	0.0369359	905	15
3	05516	W	0.100107	0.128727	0.0344158	164	16
3	05516	C	0.210285	0.13761	0.0367163	955	15
3	08026	W	0.293636	0.173373	0.0468613	973	11
3	08026	C	0.187574	0.163889	0.0433285	779	12
3	08135	W	0.281498	0.223907	0.0629609	842	8
3	08135	C	0.198122	0.128622	0.0333265	952	16
3	08233	W	0.0306077	0.0401489	0.00879667	65	60
3	08233	C	0.0312083	0.0385205	0.00879191	173	67
3	08324	W	0.130001	0.151095	0.0409121	351	13
3	08324	C	0.0967897	0.0816011	0.0199545	833	28
3	08509	W	0.189316	0.225047	0.0648768	335	8
3	08509	C	0.129469	0.125406	0.035697	650	17
3	15036	W	0.223256	0.203921	0.0590748	733	9
3	15036	C	0.163199	0.152665	0.0408646	705	13
3	15268	W	0.113042	0.151833	0.0421542	128	13
3	15268	C	0.248785	0.225787	0.0671843	722	8
3	15290	W	0.115692	0.102749	0.0275028	777	21
3	15290	C	0.0955248	0.0692983	0.015565	930	34
3	15399	W	0.134115	0.12135	0.0314217	736	17
3	15399	C	0.109014	0.0662885	0.0157374	979	36
3	16614	W	0.15114	0.0876905	0.0207863	984	25
3	16614	C	0.102012	0.131898	0.0363948	200	16
3	16684	W	0.135366	0.203612	0.0569976	43	9
3	16684	C	0.290516	0.317298	0.0961902	499	5
3	16705	W	0.29337	0.162105	0.0437922	981	12
3	16705	C	0.27373	0.377635	0.113235	178	4
3	16741	W	0.137989	0.144688	0.0398418	530	14
3	16741	C	0.167095	0.136838	0.0371045	841	15
3	16746	W	0.158679	0.121109	0.0312022	885	17
3	16746	C	0.272176	0.176903	0.0477746	952	11
3	16757	W	0.100481	0.0767234	0.0181756	904	29
3	16757	C	0.0519794	0.0665585	0.0168971	137	35
3	16765	W	0.201503	0.245143	0.0698818	302	7
3	16765	C	0.164637	0.144875	0.0388722	761	14
3	16772	W	0.0314673	0.0214768	0.00359096	984	121
3	16772	C	0.0463411	0.0251376	0.00507941	993	106
3	16788	W	0.0556525	0.0277779	0.00528946	998	89
3	16788	C	0.0717801	0.0296899	0.0062334	1000	87
3	16798	W	0.0887334	0.0665127	0.0155181	916	34
3	16798	C	0.0746403	0.0575421	0.0128867	897	41
3	16817	W	0.0523847	0.0458081	0.00969333	814	52
3	16817	C	0.0585609	0.0418498	0.00961855	936	59
3	16830	W	0.247403	0.162812	0.0459642	943	12
3	16830	C	0.139028	0.148827	0.0423823	506	14
3	16835	W	0.130277	0.106633	0.0282779	842	20
3	16835	C	0.263629	0.138116	0.0370122	992	15
3	16879	W	0.20492	0.143483	0.039062	929	14
3	16879	C	0.107115	0.117466	0.030972	449	18
3	17076	W	0.0160823	0.0133698	0.00166913	958	204
3	17076	C	0.0184426	0.0145995	0.00258743	920	199
3	46872	W	0.0282059	0.0264356	0.00475089	742	96
3	46872	C	0.0331502	0.028703	0.00600743	816	92
3	46873	W	0.280883	0.279438	0.0824004	613	6
3	46873	C	0.164473	0.131526	0.0365733	855	16

Table 13: Results for gene classes taken from level 3 of the Gene Ontology heirarchy on chromosome 3 using TIGR data with the tandem duplications removed

Chr	Class	Std	Orig. Grnwd	MC Grnwd	SD MC grnwd	Ranking	Examples
4	03677	W	0.0129923	0.0121034	0.00130761	815	192
4	03677	C	0.0169025	0.0130652	0.00182494	960	188
4	03723	W	0.0417018	0.047902	0.00884043	254	44
4	03723	C	0.0540145	0.0599444	0.0128247	361	36
4	04888	W	0.0644233	0.0888302	0.0184649	21	22
4	04888	C	0.0836343	0.0965266	0.0221236	300	21
4	05516	W	0.118923	0.149038	0.0320399	154	12
4	05516	C	0.235037	0.178224	0.0437043	924	10
4	08026	W	0.167338	0.191652	0.0510272	354	9
4	08026	C	0.287412	0.255354	0.0655557	761	6
4	08135	W	0.149115	0.161074	0.0389979	433	11
4	08135	C	0.148636	0.157594	0.0372392	465	12
4	08233	W	0.0285846	0.0324468	0.00490406	200	66
4	08233	C	0.0462363	0.0425322	0.00769399	741	52
4	08324	W	0.0989273	0.141374	0.0336064	25	13
4	08324	C	0.150823	0.105121	0.0233651	948	19
4	08509	W	0.179975	0.209712	0.0523687	328	8
4	08509	C	0.324323	0.264871	0.0720354	837	6
4	15036	W	0.181255	0.193223	0.052831	483	9
4	15036	C	0.262949	0.212784	0.0547736	851	8
4	15268	W	0.224898	0.150251	0.0353664	953	12
4	15268	C	0.163498	0.142327	0.0325918	787	13
4	15290	W	0.0952999	0.113607	0.0277873	263	17
4	15290	C	0.11193	0.1119	0.0265576	586	18
4	15399	W	0.121387	0.118155	0.0281176	647	16
4	15399	C	0.100686	0.082736	0.0189495	863	25
4	16614	W	0.0975486	0.132796	0.0320763	75	14
4	16614	C	0.116744	0.096415	0.0206631	853	21
4	16684	W	0.110852	0.139844	0.0329314	156	13
4	16684	C	0.197149	0.177635	0.0449469	752	10
4	16705	W	0.178822	0.205722	0.0504966	341	8
4	16705	C	0.303436	0.293133	0.0760366	654	5
4	16741	W	0.15553	0.140792	0.0347722	749	13
4	16741	C	0.146812	0.135301	0.0315504	699	14
4	16746	W	0.600006	0.175414	0.0410942	1000	10
4	16746	C	0.185573	0.209597	0.0520227	370	8
4	16757	W	0.0659497	0.0615748	0.0117633	736	33
4	16757	C	0.0815639	0.0685936	0.0149952	841	31
4	16765	W	0.225286	0.207875	0.0504392	725	8
4	16765	C	0.248316	0.208959	0.0514157	833	8
4	16772	W	0.0270851	0.0234177	0.00327129	892	94
4	16772	C	0.0235973	0.0271343	0.0046604	205	85
4	16788	W	0.0459036	0.0499351	0.00902391	378	42
4	16788	C	0.0611591	0.0349187	0.00629433	996	66
4	16798	W	0.0842368	0.0932126	0.0213874	400	21
4	16798	C	0.0635588	0.0585905	0.0117683	752	37
4	16817	W	0.0564313	0.045103	0.00773016	925	46
4	16817	C	0.0596262	0.0560577	0.011947	700	39
4	16830	W	0.289936	0.290194	0.0766889	583	5
4	16830	C	0.309101	0.23276	0.0586496	906	7
4	16835	W	0.168894	0.209922	0.0530881	220	8
4	16835	C	0.160676	0.109992	0.0250202	957	18
4	16879	W	0.158204	0.14082	0.0336505	761	13
4	16879	C	0.213312	0.128431	0.0304347	982	15
4	17076	W	0.0171887	0.0155604	0.00191376	843	146
4	17076	C	0.0156878	0.0171109	0.0026329	312	140
4	46872	W	0.0291204	0.0293612	0.00445817	573	73
4	46872	C	0.0268637	0.0300383	0.00534075	307	76
4	46873	W	0.159174	0.208489	0.0552013	136	8
4	46873	C	0.138872	0.156184	0.0391834	387	12

Table 14: Results for gene classes taken from level 3 of the Gene Ontology heirarchy on chromosome 4 using TIGR data with the tandem duplications removed

Chr	Class	Std	Orig. Grnwd	MC Grnwd	SD MC grnwd	Ranking	Examples
5	03677	W	0.01122	0.00813623	0.00155813	959	322
5	03677	C	0.00895194	0.0086028	0.00193718	762	321
5	03723	W	0.0517797	0.0422362	0.00833106	873	54
5	03723	C	0.0431566	0.0465661	0.00977175	447	51
5	04888	W	0.0603689	0.0591141	0.0122314	617	37
5	04888	C	0.139476	0.0932902	0.0225263	965	23
5	05516	W	0.122023	0.122446	0.0302002	586	16
5	05516	C	0.208923	0.103458	0.022912	999	20
5	08026	W	0.169112	0.131586	0.0336958	880	15
5	08026	C	0.115781	0.14224	0.0376127	238	14
5	08135	W	0.111221	0.100929	0.023443	743	20
5	08135	C	0.0835833	0.100647	0.0245403	242	21
5	08233	W	0.0240237	0.0278832	0.0053231	231	85
5	08233	C	0.0345186	0.0346533	0.00784115	613	71
5	08324	W	0.112969	0.118477	0.0314447	523	17
5	08324	C	0.106023	0.069281	0.0155078	965	32
5	08509	W	0.140147	0.170098	0.0456754	255	11
5	08509	C	0.218768	0.151233	0.0424222	922	13
5	15036	W	0.197224	0.199226	0.0533452	578	9
5	15036	C	0.137191	0.115361	0.0277765	817	18
5	15268	W	0.109074	0.15834	0.0413776	41	12
5	15268	C	0.0868651	0.08578	0.0189725	595	25
5	15290	W	0.0407779	0.0485806	0.00972985	198	46
5	15290	C	0.0580766	0.0588008	0.0131948	575	39
5	15399	W	0.0886392	0.0846687	0.0196059	673	25
5	15399	C	0.102243	0.0626415	0.0138766	981	36
5	16614	W	0.0600013	0.0739093	0.0166394	167	29
5	16614	C	0.0748891	0.0887599	0.0202041	256	24
5	16684	W	0.263056	0.307539	0.0866888	364	5
5	16684	C	0.131215	0.14284	0.0380675	459	14
5	16705	W	0.260239	0.156134	0.0396956	973	12
5	16705	C	0.167615	0.149306	0.0365214	745	13
5	16741	W	0.0936445	0.0818252	0.0199275	817	26
5	16741	C	0.11064	0.120955	0.0300102	447	17
5	16746	W	0.171211	0.12504	0.0331477	908	16
5	16746	C	0.206059	0.121051	0.0320166	979	17
5	16757	W	0.0543074	0.0594586	0.012272	382	37
5	16757	C	0.100092	0.0616616	0.0134518	988	37
5	16765	W	0.152023	0.181115	0.0522266	327	10
5	16765	C	0.161363	0.140091	0.0361292	795	14
5	16772	W	0.0214387	0.0182991	0.00314096	864	133
5	16772	C	0.0235594	0.0195708	0.00407872	880	132
5	16788	W	0.0277638	0.0287146	0.00545909	525	82
5	16788	C	0.0290335	0.0287027	0.00615458	633	87
5	16798	W	0.0906331	0.0611611	0.0133093	961	36
5	16798	C	0.0457761	0.0539012	0.0126032	276	43
5	16817	W	0.0605538	0.0351744	0.00695328	994	66
5	16817	C	0.041682	0.0314016	0.00678317	909	79
5	16830	W	0.170408	0.155443	0.0402545	746	12
5	16830	C	0.156601	0.119835	0.0285022	901	17
5	16835	W	0.145995	0.138652	0.0374562	687	14
5	16835	C	0.14122	0.161296	0.0455584	389	12
5	16879	W	0.245606	0.171062	0.0489063	920	11
5	16879	C	0.0937029	0.113763	0.0281602	237	18
5	17076	W	0.0174472	0.0104488	0.00191839	987	244
5	17076	C	0.0161141	0.0111795	0.0015269	1000	257
5	46872	W	0.0211967	0.0168163	0.00295392	927	146
5	46872	C	0.0202159	0.0216254	0.00435813	432	118
5	46873	W	0.140386	0.170474	0.0453658	272	11
5	46873	C	0.16261	0.131026	0.0320155	870	15

Table 15: Results for gene classes taken from level 3 of the Gene Ontology heirarchy on chromosome 5 using TIGR data with the tandem duplications removed

Chr	Class	Std	Orig. Grnwd	MC Grnwd	SD MC grnwd	Ranking	Examples
1	04175	W	0.0513475	0.0562593	0.00995227	345	37
1	04175	W	0.0513475	0.0563434	0.0101552	350	37
1	04175	C	0.0449739	0.0478894	0.00929813	444	46
1	04175	W	0.0513475	0.05688	0.0106956	340	37
1	04175	W	0.0513475	0.0557699	0.00967406	361	37
1	04175	C	0.0449739	0.0475833	0.00890772	445	46
1	04518	W	0.109605	0.0745582	0.014036	972	27
1	04518	C	0.0995586	0.0883178	0.0189466	783	23
1	04553	W	0.0557008	0.0538762	0.00932359	648	39
1	04553	C	0.0374352	0.0410777	0.00773694	355	54
1	04930	W	0.108466	0.0990071	0.0226273	747	20
1	04930	C	0.0739106	0.0846932	0.0180843	310	24
1	05216	W	0.304123	0.212347	0.0604464	920	8
1	05216	C	0.19828	0.156097	0.0403301	877	12
1	05351	W	0.15101	0.16419	0.042401	452	11
1	05351	C	0.232256	0.103538	0.0229448	999	19
1	05509	W	0.0679089	0.060738	0.0110737	791	34
1	05509	C	0.125761	0.0705349	0.0142229	994	30
1	08168	W	0.0598148	0.0902619	0.0196243	1	22
1	08168	C	0.0757883	0.0931364	0.020261	166	22
1	08234	W	0.140658	0.118072	0.0264722	838	16
1	08234	C	0.0641682	0.0727113	0.0149595	307	29
1	08236	W	0.141556	0.0963499	0.0194063	971	20
1	08236	C	0.0781281	0.0852379	0.0184905	413	24
1	08237	W	0.0953594	0.120607	0.0299271	151	16
1	08237	C	0.118285	0.0995007	0.0212722	839	20
1	08238	W	0.142008	0.134512	0.0342668	680	14
1	08238	C	0.136302	0.144898	0.0382489	508	13
1	15077	W	0.182286	0.152211	0.0353606	838	12
1	15077	C	0.126123	0.128138	0.0306994	572	15
1	15291	W	0.0543088	0.0471359	0.00802181	844	45
1	15291	C	0.0558487	0.0453071	0.00905796	903	49
1	15405	W	0.09005	0.0586559	0.0111785	983	36
1	15405	C	0.0855052	0.083012	0.0174757	624	25
1	16301	W	0.0241956	0.0144615	0.00181786	999	162
1	16301	C	0.0261603	0.0159654	0.00235532	998	153
1	16616	W	0.109443	0.0724273	0.0135826	985	28
1	16616	C	0.0929066	0.0859	0.0191422	742	24
1	16747	W	0.141099	0.117739	0.027828	846	16
1	16747	C	0.0925472	0.0768066	0.0153089	850	27
1	16758	W	0.105398	0.0868072	0.0190894	867	23
1	16758	C	0.0805193	0.0593405	0.0119754	949	36
1	16773	W	0.0265585	0.0175763	0.00232149	995	130
1	16773	C	0.0307395	0.021999	0.00375021	970	108
1	16779	W	0.101478	0.0779168	0.0152251	923	26
1	16779	C	0.0827133	0.0792753	0.0169494	670	26
1	16789	W	0.116849	0.0808929	0.0164428	961	25
1	16789	C	0.0929107	0.0799874	0.017765	825	26
1	16818	W	0.0420057	0.0443335	0.0074675	439	49
1	16818	C	0.0471878	0.0373437	0.00644888	923	60
1	16820	W	0.1189	0.0783641	0.0149581	982	26
1	16820	C	0.120898	0.110661	0.0271952	732	18
1	16836	W	0.126712	0.153157	0.0407151	258	12
1	16836	C	0.214194	0.155979	0.0428392	910	12
1	16881	W	0.138938	0.143282	0.037581	550	13
1	16881	C	0.283063	0.216109	0.0593664	877	8
1	19001	W	0.0758917	0.0769395	0.0155041	546	26
1	19001	C	0.0801111	0.0646556	0.0134908	882	33
1	30554	W	0.0116206	0.0106348	0.00124603	823	225
1	30554	C	0.0131895	0.00942394	0.00107213	994	275
1	42578	W	0.0626789	0.0600429	0.011554	665	35
1	42578	C	0.0492096	0.0595325	0.0117279	177	36
1	46914	W	0.0335562	0.0258415	0.00373338	962	86
1	46914	C	0.0269117	0.0240134	0.00383419	808	97

Table 16: Results for gene classes taken from level 4 of the Gene Ontology heirarchy on chromosome 1 using TIGR data with the tandem duplications removed

Chr	Class	Std	Orig. Grnwd	MC Grnwd	SD MC grnwd	Ranking	Examples
2	04175	W	0.130923	0.158283	0.0391246	266	12
2	04175	C	0.0633672	0.0832933	0.0176976	83	25
2	04518	W	0.0632686	0.067484	0.0147448	462	32
2	04518	C	0.0995639	0.0788869	0.0182116	888	27
2	04553	W	0.10141	0.0959823	0.0237089	696	22
2	04553	C	0.137122	0.0846905	0.0209282	970	25
2	04930	W	0.216652	0.166296	0.0408891	900	11
2	04930	C	0.232543	0.16621	0.0395209	933	11
2	05216	W	0.184078	0.19591	0.0494723	479	9
2	05216	C	0.386627	0.294625	0.0770053	894	5
2	05351	W	0.248186	0.266352	0.0700611	475	6
2	05351	C	0.119458	0.166081	0.0400158	69	11
2	05509	W	0.130467	0.109584	0.0269941	826	19
2	05509	C	0.114574	0.118733	0.0302282	539	17
2	08168	W	0.20979	0.19595	0.0497701	677	9
2	08168	C	0.160775	0.16835	0.0429306	515	11
2	08234	W	0.458523	0.169967	0.0407021	999	11
2	08234	C	0.0655755	0.0729867	0.0155429	378	29
2	08236	W	0.189919	0.193229	0.0483657	564	9
2	08236	C	0.137261	0.147925	0.0382315	479	13
2	08237	W	0.12911	0.123147	0.0295054	664	16
2	08237	C	0.106069	0.129361	0.0302272	232	15
2	08238	W	0.364146	0.235382	0.0625365	961	7
2	08238	C	0.118795	0.154172	0.036845	130	12
2	15077	W	0.135514	0.158841	0.0405254	312	12
2	15077	C	0.181782	0.261148	0.0669092	49	6
2	15291	W	0.105731	0.0940487	0.023445	782	22
2	15291	C	0.0749605	0.0681076	0.015131	741	32
2	15405	W	0.117556	0.0970187	0.0221987	848	21
2	15405	C	0.142166	0.107537	0.0254887	891	19
2	16301	W	0.0291628	0.0267498	0.00448018	788	86
2	16301	C	0.0467824	0.0233949	0.00390525	999	100
2	16616	W	0.201552	0.146344	0.0353168	924	13
2	16616	C	0.172182	0.167402	0.0409736	642	11
2	16747	W	0.109136	0.137495	0.0312844	180	14
2	16747	C	0.0926203	0.106723	0.0251298	331	19
2	16758	W	0.0754195	0.0836011	0.0192207	397	25
2	16758	C	0.179883	0.131982	0.0333749	907	15
2	16773	W	0.0354505	0.03743	0.00784033	504	61
2	16773	C	0.0654902	0.029929	0.00546081	1000	77
2	16779	W	0.0429206	0.052901	0.0114393	154	42
2	16779	C	0.052084	0.0485073	0.00992722	735	46
2	16789	W	0.13646	0.0970424	0.0217254	930	21
2	16789	C	0.188477	0.155326	0.0396634	842	12
2	16818	W	0.0517624	0.0526443	0.0107515	564	42
2	16818	C	0.0827753	0.0713228	0.0161384	821	30
2	16820	W	0.139369	0.125466	0.0288928	735	16
2	16820	C	0.217831	0.167474	0.040854	891	11
2	16836	W	0.206834	0.165491	0.0399289	864	11
2	16836	C	0.20676	0.179301	0.0425763	781	10
2	16881	W	0.264655	0.183735	0.047894	942	10
2	16881	C	0.243628	0.179358	0.0422828	931	10
2	19001	W	0.119487	0.132036	0.0327903	414	15
2	19001	C	0.11199	0.111385	0.0266912	621	18
2	30554	W	0.0125171	0.0153304	0.00214348	46	156
2	30554	C	0.0166748	0.015771	0.00235116	726	151
2	42578	W	0.166253	0.12504	0.0299752	903	16
2	42578	C	0.144693	0.0767527	0.018392	989	28
2	46914	W	0.0598407	0.0366249	0.00757244	982	62
2	46914	C	0.073084	0.0421166	0.00823319	991	53

Table 17: Results for gene classes taken from level 4 of the Gene Ontology heirarchy on chromosome 2 using TIGR data with the tandem duplications removed

Chr	Class	Std	Orig. Grnwd	MC Grnwd	SD MC grnwd	Ranking	Examples
3	04175	W	0.0828367	0.0961691	0.0232794	330	22
3	04175	C	0.0582969	0.0765958	0.0172947	92	30
3	04518	W	0.0949133	0.0869089	0.0207863	740	25
3	04518	C	0.181452	0.15358	0.0414696	800	13
3	04553	W	0.0921558	0.0673205	0.0149732	936	33
3	04553	C	0.0837389	0.0658764	0.0161823	872	36
3	04930	W	0.184884	0.141904	0.0363811	886	14
3	04930	C	0.205915	0.155036	0.0419373	894	13
3	05216	W	0.154022	0.205372	0.0581426	158	9
3	05216	C	0.482657	0.322519	0.098263	925	5
3	05351	W	0.278424	0.246291	0.0697688	740	7
3	05351	C	0.255614	0.164705	0.0466587	945	12
3	05509	W	0.0729502	0.0832001	0.0195152	341	26
3	05509	C	0.0812411	0.090279	0.0237967	430	25
3	08168	W	0.137989	0.142825	0.0374344	559	14
3	08168	C	0.167222	0.145615	0.039742	779	14
3	08234	W	0.0722828	0.110967	0.0300086	12	19
3	08234	C	0.108186	0.117789	0.0295892	449	18
3	08236	W	0.147753	0.141818	0.0379138	663	14
3	08236	C	0.195742	0.145048	0.0383188	908	14
3	08237	W	0.179706	0.161171	0.0455517	746	12
3	08237	C	0.228625	0.176214	0.0493472	868	11
3	08238	W	0.177507	0.162559	0.047588	718	12
3	08238	C	0.279828	0.146712	0.0420409	987	14
3	15077	W	0.25912	0.247725	0.0680434	654	7
3	15077	C	0.230877	0.207613	0.0585072	731	9
3	15291	W	0.115692	0.101581	0.0278023	783	21
3	15291	C	0.0955248	0.0690029	0.0164043	925	34
3	15405	W	0.135881	0.135328	0.0379315	624	15
3	15405	C	0.112491	0.0720566	0.0182815	966	33
3	16301	W	0.0434729	0.0212459	0.00384822	999	124
3	16301	C	0.0424516	0.0230334	0.00454562	994	117
3	16616	W	0.155618	0.110908	0.0281267	920	19
3	16616	C	0.107058	0.138254	0.0370709	180	15
3	16747	W	0.162427	0.12691	0.0294896	887	16
3	16747	C	0.290047	0.208166	0.0595817	912	9
3	16758	W	0.157957	0.115376	0.0294689	912	18
3	16758	C	0.0819283	0.0999651	0.0257017	264	22
3	16773	W	0.0449781	0.0257899	0.00488935	997	98
3	16773	C	0.048904	0.0325901	0.00706568	967	80
3	16779	W	0.147724	0.121613	0.0309004	847	17
3	16779	C	0.135605	0.130562	0.0337814	637	16
3	16789	W	0.0941813	0.0721643	0.0170446	914	31
3	16789	C	0.120314	0.0892498	0.0219505	911	25
3	16818	W	0.0641669	0.0622456	0.0145049	658	36
3	16818	C	0.0628488	0.0493296	0.0112963	884	50
3	16820	W	0.226331	0.204136	0.0557434	735	9
3	16820	C	0.133456	0.0896865	0.0219195	964	25
3	16836	W	0.194999	0.150641	0.0417673	864	13
3	16836	C	0.484423	0.229455	0.0672273	990	8
3	16881	W	0.207774	0.186377	0.0529435	730	10
3	16881	C	0.166702	0.165997	0.0460357	610	12
3	19001	W	0.197903	0.131932	0.0325161	954	15
3	19001	C	0.192229	0.110521	0.0294331	981	20
3	30554	W	0.0162237	0.0141116	0.00208803	880	198
3	30554	C	0.0185255	0.0151088	0.0027518	898	191
3	42578	W	0.109951	0.0567932	0.0124646	997	40
3	42578	C	0.0882181	0.0592558	0.0142728	955	40
3	46914	W	0.0405973	0.0352331	0.00700902	828	69
3	46914	C	0.0577903	0.0420076	0.00890319	943	60

Table 18: Results for gene classes taken from level 4 of the Gene Ontology heirarchy on chromosome 3 using TIGR data with the tandem duplications removed

Chr	Class	Std	Orig. Grnwd	MC Grnwd	SD MC grnwd	Ranking	Examples
4	04175	W	0.0809505	0.0859348	0.0179553	472	23
4	04175	C	0.0903446	0.116667	0.0293574	127	17
4	04518	W	0.130448	0.163497	0.0415675	187	11
4	04518	C	0.14075	0.154779	0.0353639	394	12
4	04553	W	0.0978304	0.0976322	0.024293	628	20
4	04553	C	0.0641654	0.0596606	0.0117898	730	36
4	04930	W	0.0852606	0.132234	0.0334619	7	14
4	04930	C	0.114211	0.163782	0.0407241	34	11
4	05216	W	0.282537	0.210099	0.0537041	908	8
4	05216	C	0.378377	0.194926	0.0498971	992	9
4	05351	W	0.185814	0.258021	0.0673413	75	6
4	05351	C	0.193436	0.235765	0.0626581	248	7
4	05509	W	0.0643465	0.105155	0.0235894	0	18
4	05509	C	0.110167	0.10543	0.0250035	689	19
4	08168	W	0.15553	0.142188	0.0357029	742	13
4	08168	C	0.146812	0.135734	0.0302899	693	14
4	08234	W	0.0870523	0.101719	0.0214013	264	19
4	08234	C	0.0955579	0.0905114	0.0200065	686	23
4	08236	W	0.175203	0.162106	0.0395191	708	11
4	08236	C	0.156771	0.137068	0.0334364	775	14
4	08237	W	0.0932976	0.100795	0.0224498	439	19
4	08237	C	0.176593	0.259332	0.0670985	32	6
4	08238	W	0.124098	0.111082	0.0252676	769	17
4	08238	C	0.271337	0.233592	0.0573532	800	7
4	15077	W	0.210815	0.294016	0.0815613	80	5
4	15077	C	0.228909	0.165362	0.0389922	930	11
4	15291	W	0.0952999	0.112246	0.0241634	262	17
4	15291	C	0.11193	0.112212	0.0286301	592	18
4	15405	W	0.122235	0.123266	0.0265472	569	15
4	15405	C	0.103456	0.0926053	0.0198511	763	22
4	16301	W	0.0337529	0.0238267	0.00324817	987	91
4	16301	C	0.0284825	0.0258301	0.00453163	793	90
4	16616	W	0.0987236	0.139429	0.0321978	45	13
4	16616	C	0.131379	0.109607	0.0247594	845	18
4	16747	W	0.608741	0.229102	0.0620424	1000	7
4	16747	C	0.312581	0.231202	0.0601837	919	7
4	16758	W	0.0892111	0.107639	0.0254762	232	18
4	16758	C	0.108081	0.0997762	0.0216606	712	20
4	16773	W	0.0472746	0.0328362	0.00528212	981	66
4	16773	C	0.0396203	0.036579	0.00688912	759	62
4	16779	W	0.0808354	0.0886275	0.0177636	379	22
4	16779	C	0.0886001	0.122214	0.0282606	51	16
4	16789	W	0.196734	0.159638	0.0390374	867	11
4	16789	C	0.130747	0.121507	0.0281815	699	16
4	16818	W	0.0707813	0.0636646	0.0121313	770	32
4	16818	C	0.0999115	0.0801637	0.0163089	887	26
4	16820	W	0.294318	0.206485	0.0526063	933	8
4	16820	C	0.17155	0.120918	0.0272221	951	16
4	16836	W	0.28153	0.25623	0.0713785	730	6
4	16836	C	0.174081	0.145736	0.0346181	823	13
4	16881	W	0.284257	0.298884	0.0815264	535	5
4	16881	C	0.252904	0.151656	0.0347388	981	12
4	19001	W	0.0891474	0.0934613	0.0214568	515	21
4	19001	C	0.159834	0.144245	0.0326452	748	13
4	30554	W	0.0175082	0.0160527	0.0019055	809	142
4	30554	C	0.0165178	0.0178253	0.00315113	402	135
4	42578	W	0.0751362	0.101486	0.0225922	56	19
4	42578	C	0.121264	0.0733666	0.015393	986	29
4	46914	W	0.0469888	0.0406314	0.00644743	855	52
4	46914	C	0.0415339	0.0428242	0.0079099	511	52

Table 19: Results for gene classes taken from level 4 of the Gene Ontology heirarchy on chromosome 4 using TIGR data with the tandem duplications removed

Chr	Class	Std	Orig. Grnwd	MC Grnwd	SD MC grnwd	Ranking	Examples
5	04175	W	0.0602489	0.0578787	0.0120805	653	39
5	04175	C	0.049675	0.0620271	0.0137616	163	37
5	04518	W	0.0794314	0.0924232	0.0228543	310	23
5	04518	C	0.0785277	0.103793	0.0255301	93	20
5	04553	W	0.0927926	0.0657474	0.0137978	952	33
5	04553	C	0.0488214	0.0572722	0.012288	254	40
5	04930	W	0.0925231	0.117402	0.0302261	182	17
5	04930	C	0.174387	0.201923	0.0559239	360	9
5	05216	W	0.129782	0.184684	0.0522799	61	10
5	05216	C	0.106138	0.096063	0.022006	762	22
5	05351	W	0.162189	0.138181	0.0345115	815	14
5	05351	C	0.205858	0.243485	0.0697066	346	7
5	05509	W	0.0671803	0.0673656	0.0146473	604	32
5	05509	C	0.0688808	0.0751554	0.0160773	404	29
5	08168	W	0.0936445	0.0827125	0.0185907	771	26
5	08168	C	0.11064	0.118881	0.0275203	455	17
5	08234	W	0.0963299	0.100237	0.0258597	546	21
5	08234	C	0.109508	0.153285	0.0423915	89	13
5	08236	W	0.0787365	0.076426	0.0172648	648	28
5	08236	C	0.106795	0.0959824	0.0233556	746	22
5	08237	W	0.135089	0.112475	0.0284624	826	18
5	08237	C	0.137204	0.111444	0.0268743	864	19
5	08238	W	0.172127	0.148829	0.0381274	803	13
5	08238	C	0.143806	0.151473	0.0415583	523	13
5	15077	W	0.179947	0.201872	0.0586895	431	9
5	15077	C	0.191129	0.120371	0.0301801	971	17
5	15291	W	0.0407779	0.0488679	0.0105603	202	46
5	15291	C	0.0580766	0.0585064	0.012613	564	39
5	15405	W	0.108183	0.102527	0.0240083	674	20
5	15405	C	0.10619	0.0783856	0.0175298	936	28
5	16301	W	0.0306068	0.018271	0.00324143	992	133
5	16301	C	0.0220163	0.0173014	0.00340377	910	151
5	16616	W	0.0801271	0.0833716	0.0183193	503	25
5	16616	C	0.0957847	0.100318	0.0237078	514	21
5	16747	W	0.174295	0.140148	0.0360842	860	14
5	16747	C	0.209899	0.133287	0.0345469	970	15
5	16758	W	0.116678	0.0937626	0.0214066	885	22
5	16758	C	0.20706	0.0861903	0.0188635	1000	25
5	16773	W	0.0374379	0.0236634	0.00432713	992	101
5	16773	C	0.0251962	0.0227385	0.00478526	781	112
5	16779	W	0.0791245	0.0899414	0.0204316	337	23
5	16779	C	0.14305	0.118783	0.0294427	828	17
5	16789	W	0.0927072	0.0763825	0.0170208	850	28
5	16789	C	0.0679968	0.0860694	0.019992	134	25
5	16818	W	0.067962	0.0487938	0.00959897	956	46
5	16818	C	0.0499645	0.0406861	0.00890985	867	59
5	16820	W	0.144629	0.146245	0.0387674	599	13
5	16820	C	0.113412	0.0921847	0.0211511	866	23
5	16836	W	0.152897	0.169911	0.045352	415	11
5	16836	C	0.191063	0.223132	0.065604	356	8
5	16881	W	0.389263	0.30803	0.0899876	837	5
5	16881	C	0.1838	0.17098	0.0478417	704	11
5	19001	W	0.0920855	0.074159	0.0167015	876	29
5	19001	C	0.138827	0.0829745	0.0194841	984	26
5	30554	W	0.0176474	0.0106649	0.00150047	998	235
5	30554	C	0.0162879	0.0107516	0.00193222	980	250
5	42578	W	0.0690198	0.0755282	0.0161162	392	28
5	42578	C	0.0603234	0.0627738	0.0136233	515	36
5	46914	W	0.0358101	0.0241335	0.00444273	979	99
5	46914	C	0.0382661	0.0304407	0.00663895	880	82

Table 20: Results for gene classes taken from level 4 of the Gene Ontology heirarchy on chromosome 5 using TIGR data with the tandem duplications removed
